# Mapping dynamic working life patterns and the impact of occupational exposures: a scoping review

**DOI:** 10.1186/s12889-025-23487-5

**Published:** 2025-07-03

**Authors:** Rachel Louise Hasting, Karen Marieke Oude Hengel, Taina Leinonen, Ute Bültmann, Ingrid Sivesind Mehlum, Alex Burdorf, Michelle C Turner, Laura Salonen, Damien M McElvenny, Svetlana Solovieva

**Affiliations:** 1 National Institute of Occupational Health, Pb 5330 Majorstuen, Oslo, 0304 Norway; 2 Department of Work, Health and Technology, TNO, Leiden, The Netherlands; 3 Finnish Institute of Occupational Health, Helsinki, Finland; 4 Department of Community and Occupational Medicine, University of Groningen, University Medical Center Groningen, Groningen, The Netherlands; 5 Department of Occupational and Environmental Medicine, Copenhagen University Hospital - Bispebjerg and Frederiksberg, Copenhagen, Denmark; 6 Department of Public Health, University of Copenhagen, Copenhagen, Denmark; 7 Department of Public Health, Erasmus MC, Rotterdam, The Netherlands; 8 Barcelona Institute for Global Health (ISGlobal), Barcelona, Spain; 9 Universitat Pompeu Fabra (UPF), Barcelona, Spain; 10 CIBER Epidemiología y Salud Pública (CIBERESP), Madrid, Spain; 11 Institute of Occupational Medicine, Edinburgh, UK; 12 University of Manchester, Manchester, UK

**Keywords:** Working life expectancy, Working life patterns, Work disability, Sickness absence, Work participation, Occupational exposure

## Abstract

**Background:**

An ageing population and increasing life expectancy has intensified pressure to prolong working lives among high-income countries. Emerging research has sought to characterise dynamic working life patterns (how labour market participation changes over the working career), and how various factors, including occupational exposures, influence these patterns. This scoping review aims to systematically map the literature in this area and to identify future research needs.

**Methods:**

A systematic search was carried out in PubMed, Embase, Web of Science and Scopus. Original studies were included if they included individuals from the general working-age population (defined as 18–70 years of age) or from patient-, sector-, industry-, or occupation-specific populations, and if they examined associations between at least one occupational exposure and a measure of dynamic working life patterns, grouped into either labour market participation trajectories or cumulative time spent in various labour market states. Studies were considered too heterogeneous to allow for quantitative synthesizing of results or calculation of an average measure of working life patterns across studies by exposure.

**Results:**

The seventeen included original studies were heterogenous with regards to study populations, analysis methods, occupational exposures, and outcomes. Studies of biomechanical and psychosocial exposures were the most common, with indications that biomechanical factors are associated with reduced work participation.

**Conclusions:**

Future studies would benefit from clearer definitions of occupational exposures and measures of dynamic working life patterns, a broader inclusion of occupational exposures, and measures of cumulative exposure.

**Supplementary Information:**

The online version contains supplementary material available at 10.1186/s12889-025-23487-5.

## Background

Due to an ageing population and increasing life expectancy in high income countries, the old-dependency ratio (number of individuals aged 65 years or older per 100 people of working age) has substantially increased in OECD countries from 20% in 1975 towards 34% in 2024 [1]. The increased dependency ratio means that pension and care costs will increase, whilst fewer individuals are available to work to fund these increased costs. Many countries have responded to this public finance issue by implementing policies and measures aimed at increasing work participation and prolonging working lives [2]. Labour force participation has in turn increased in many OECD countries, with the sharpest increase in the age group 55–64 years, from 50% in 2000 towards 66% in 2023 [3].

Individuals’ working lives can be dynamic over time, meaning they may have periods with and without paid employment throughout their working lives [4]. Further insight into how different factors, policies and measures influence these working life patterns is needed. This is hard to capture using a traditional approach of investigating one exposure and one outcome (e.g., onset of sickness absence). To date, two main approaches exist which capture working life patterns: labour market participation trajectories and cumulative time spent in different labour market states. Labour market participation trajectories map patterns in employment, work disability (reduced work participation due to health-related reasons, either temporary or permanent), unemployment, and/or other non-employment states over time during the working career. Individuals with similar patterns are then grouped together. Cumulative time spent in labour market states is often examined in terms of working life expectancy (WLE) or working years lost (WYL). WLE is a measure of the expected number of years spent in paid employment after a given age [4–6]. WYL reflects the number of working years lost due to not being in paid employment, which can be broken down into specific causes, such as work disability, unemployment or early retirement. These dynamic approaches offer an opportunity to study the influence of occupational exposures on labour market participation, taking into account the changing nature of individuals’ labour market participation over their working lives.

In recent decades, research has been conducted into whether dynamic working life patterns differ depending on sociodemographic (i.e. educational level, gender) or health-related factors. A recent scoping review indicated that a higher educational level is associated with a higher WLE compared to a lower educational level, more specifically because of fewer WYL due to unemployment and disability pension [7]. In line with this, another study including over a million individuals in Finland found that those with lower income or that held manual occupations were more likely to be clustered into a trajectory with a premature, permanent exit from work [8]. Though disease-specific information is scarce, some health outcomes, such as depression and chronic musculoskeletal pain, were related to more WYL [9]. Unhealthy WLE, whereby individuals work whilst in suboptimal health (as opposed to healthy WLE (HWLE)), has increased in all 14 OECD countries in the period 2002–2017 [10].

In addition to sociodemographic and health-related factors, it is important to acknowledge that occupational exposures also affect labour market participation. Well-known examples of this include the association of physically demanding work and emotional demands with increased disability pension [11, 12]. At present, these associations have mainly been studied using traditional methods, measuring occupational exposures one at a time or in a grouped variable and exploring associations of this exposure measure, often only measured at one point in time, with a subsequent static labour market participation outcome. Research on more dynamic working life patterns has emerged in more recent years [13, 14]. A comprehensive overview of the existing research on the role of occupational exposures in working life patterns is needed to synthesise the existing knowledge, identify knowledge gaps, and provide recommendations for future research.

## Methods

### Design

A scoping review methodology was adopted to answer the aims of the study due to the heterogeneous outcomes and research questions [15]. This scoping review was guided by the framework of Arksey and O’Malley [16].

### Identifying the research questions

The research questions were as follows: [1] what knowledge is available on the associations between occupational exposures and dynamic working life patterns, and, reflecting on the findings of the scoping review [2], what should future research on occupational exposure and working life patterns prioritise moving forward?

### Search strategy

We conducted a comprehensive search of published peer-reviewed original studies without applying restrictions on language or publication year, using PubMed, Embase, Web of Science and Scopus. The first searches were conducted on the 24th March 2023, and additional searches to update the original search were conducted on the 22nd April 2024 and the 27th January 2025. A list of keywords used can be found in Supplementary Table S1. All searches can be found in Supplementary Table S2. Reference lists of all included studies were hand-searched for potentially relevant studies.

### Study selection

For inclusion, original studies had to include either individuals from the general working-age population (defined as 18–70 years of age) or from patient-, sector-, industry-, or occupation-specific populations (see Supplementary Table S3 for screening guidelines). At least one occupational exposure had to be examined, though exposures could be grouped, for example into biomechanical (e.g., heavy physical work, awkward posture, force), psychosocial working conditions (e.g., job strain, which consists of low job autonomy and high job demands), and physical and chemical work environment (e.g., occupational exposure to noise, dust, chemicals). The outcomes of interest in this scoping review were dynamic working life patterns, defined as labour market participation trajectories or cumulative time spent in different labour market states (such as WLE, WYL, and HWLE). Studies that included only static labour market participation outcomes (e.g., employment status or onset of work disability) were excluded.

All papers retrieved from the search were uploaded to the online Covidence systematic review software [17], where duplicates were removed. RLH, KOH, UB, DMM, and SS participated in the initial screening, where at least two independent researchers screened studies on inclusion and exclusion criteria. Subsequently, the full texts of potentially relevant studies were obtained and further screened by RLH, KOH, TL, and SS, using the inclusion and exclusion criteria (Supplementary Table 3). In both stages, conflicts were discussed and resolved among authors.

### Charting the data

A data extraction template was adapted for this review (see Supplementary Table S4 for the list of headings and description of extracted information). The form was piloted on three randomly selected articles before full data extraction. If multiple papers were from the same study source, the reviewers checked whether the study results were duplicated. Data extraction was conducted by RHL, KOH, DMM and SS. One reviewer completed the extraction, and a second reviewer independently checked the extracted data. Disagreements were discussed and resolved.

### Collating, summarizing, and reporting results

A descriptive table of all included studies was created, based on the data extraction form (Table 1), along with a table that grouped studies by outcome (Table 2). A more comprehensive summary of study findings is available in Supplementary Table S5. Studies were considered too heterogeneous to allow for quantitative synthesizing of results or calculation of an average measure of working life patterns across studies by exposure. As the purpose of the review is to map the available evidence, rather than evaluate the available evidence, no assessment of methodological quality was performed.

## Results

### Literature search and exclusion of studies

**Fig. 1:**
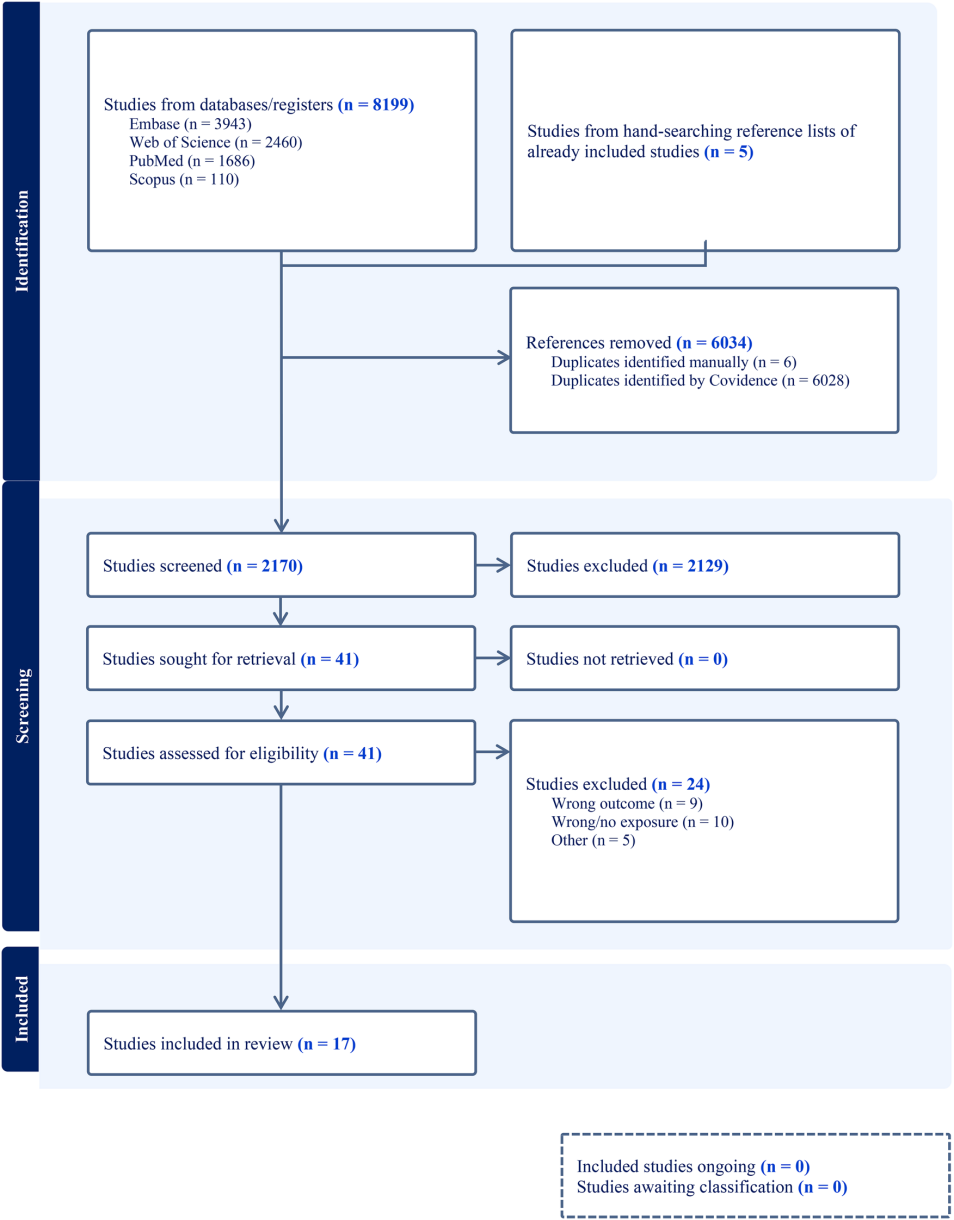
PRISMA diagram for the literature search and exclusion of studies

The primary searches in databases and registers resulted in a total of 8199 studies identified, with a further 5 studies identified from hand-searching the reference lists of already included studies. Of these, 6034 were identified as duplicates. Of the 2170 original unique studies included in the initial title and abstract screening, 41 studies were full text screened. Finally, a total of 17 original studies were included in this scoping review (see Fig. 1 for the PRISMA diagram).

### Overall study characteristics

Sixteen of the 17 studies were published between 2019 and 2024, whilst one study was published in 2013 [18] (see Table 1). Most studies (*n* = 14) were conducted in Nordic countries (Finland, Sweden, and Denmark). One study was carried out in the Netherlands [19], one study in the United Kingdom [20], and one study in the USA [21]. Two studies from Finland used the same survey, though with different study aims [18, 22]. The smallest study included 981 participants [23] and the largest study included 2,187,630 participants [24]. Nine studies included the general working population [18–20, 22, 24–29], whereas the seven other studies included a specific sector or work population. Two of these seven studies included workers from the public sector [30, 31], two studies included industry-specific workers (trade and retail and manufacturing) [21, 32], one study included workers in cleaning, manufacturing, and transportation [23], one study included individuals attending vocational rehabilitation [33], and one study included private and public sector employees receiving partial sickness benefits [34]. Three of the 17 studies used a full working age range of either 18–67 years [32], 18–65 years [27], or 18–64 years [26]; the other studies used narrower age ranges. Five studies [19, 20, 28, 29, 31] focused exclusively on older workers aged 50 years and above, and two studies did not specify the age range included [21, 23]. Nine studies considered men and women separately in the analyses [20, 24–31]. Follow-up time varied across the studies, with the shortest follow-up time at 1 year [23] and the longest at 17 years [21]. The modelled length of time for the estimation of WLE/WYL/HWLE or working days lost varied from 2 years [26] to a maximum of 30 years [27].

**Table 1:** Characteristics of the included studies

Author, year (ref)	Study sample and setting	Time period	Data source/specific cohort or study	Occupational exposure(s), type of measure	Working life pattern outcome (states)	Measurement, unit
*Working life patterns based on work disability*						
Hallman et al. 2019 [23]	*N* = 981 blue- and white-collar workers (51% female); Denmark	1 year (baseline 2012–2014)	Danish Physical Activity Cohort with Objective measurements (DPHACTO)	Biomechanical (physical exertion at work, lifting and carrying, pushing/pulling)	SA due to musculoskeletal diseases; self-reported	Trajectories (latent class growth analysis); measured in days
				Psychosocial (decision authority)		
				Self-reported, measured at baseline		
Haukka et al. 2013 [18]	*N* = 3 420 individuals aged 30–55, employed year preceding baseline (52% female); Finland	7 years (2001–2008)	Health 2000 survey, linked to national administrative registries	Biomechanical (heavy physical work involving lifting and carrying)	SA; registry-based	Trajectories (Group-Based Trajectory Modelling); measured in days
				Psychosocial (job demands, job control, supervisor support, co-worker support)		
				Self-reported at baseline, current and past jobs		
Lallukka et al. 2019 [22]	*N* = 3 814 individuals aged 30–59 and employed at baseline (53% female); Finland	7 years (2001–2008)	Health 2000 Survey, linked to national administrative registries	Biomechanical (strenuous physical work, frequent handling of loads > 5 kg, manual handling of loads > 20 kg, squatting or kneeling, working in bent postures, working with the arms above shoulder level, work requiring high handgrip force, repetitive arm movement, prolonged standing or walking, prolonged sitting, prolonged keyboard use, use of a vibrating tool)	SA; registry-based	Trajectories (Group-Based Trajectory Modelling); measured yearly
				*Combined variables*: Factors that could decrease the risk of SA (sitting and computer work combined)		
				Number of factors that could increase the risk of SA, i.e., exposure to the other nine factors, further classified into four groups: 0, 1, 2–3 or 4 or more work-related exposures.		
				Self-reported in 2001 for prior 15 years		
Leino-Arjas et al. 2021 [30]	*N* = 6 257 municipal workers aged 44–58 at baseline (53% female); Finland.	15 years (1981–1996)	Finnish Longitudinal Study of Ageing Municipal Employees (FLAME), linked to national administrative registries	Biomechanical (awkward work postures (bent or twisted postures, otherwise poor posture, repetitive movements), physical demands (carrying and standing, frequent movement))	SA; registry-based	Trajectories (Group-Based Trajectory Modelling); measured in days
				Psychosocial (job control, mental demands)		
				Physical and chemical work exposures (dirtiness (dust, smoke, steam, etc.,), risk of accident, noise, vibration, lighting and glare, heat, cold, and changing temperature, dryness, restless environment and noisy people)		
				Self-reported at baseline		
Farrants & Alexanderson 2022 [32]	*N* = 189 321 privately employed white-collar workers in trade and retail aged 18–67 and employed at baseline (44% female); Sweden	7 years (2010–2016)	National administrative registries	Psychosocial (job demands, job control)	Sickness absence (SA), disability pension (DP); registry-based	Trajectories (Group-Based Trajectory Modelling); measured in years
				JEM, connected to occupation in 2012		
Salonen et al. 2020 [24]	*N* = 2 187 630 individuals aged 30–54 and employed at baseline (49% female); Sweden	11 years (2001–2012)	National administrative registries	Psychosocial (job demands, job control)	SA, DP; registry-based	Trajectories (Group-Based Trajectory Modelling); measured in days
				JEM connected to occupation at baseline (2001)		
Shiri et al. 2021 [31]	*N* = 1 630 employees of the city of Helsinki aged 55 at baseline (81% female); Finland	10 years (baseline 2000–2002)	Finnish Helsinki Health Study, linked to personnel register of City of Helsinki	Biomechanical (heavy lifting, back rotations, awkward working positions, repetitive movements, vibration, standing, walking, sitting)	SA, DP; registry-based	Number of days lost (predictors calculated by negative binomial regression)
				Psychosocial (job strain)		
				Self-reported at baseline		
*Working life patterns based on multiple states*,* trajectories*						
Gémes et al. 2023 [25]	*N* = 9 269 individuals aged 18–50 and employed at baseline (49% female); Sweden	15 years (baseline 2000–2003)	Swedish Living Conditions Surveys, linked to national administrative registries	Biomechanical (physically strenuous job)	Employment, unemployment, SA, DP, parental leave; registry-based	Sequences (weighted cluster analysis); measured in years
				Psychosocial (mentally strenuous job, monotonous job, possibility to learn new things at work, hectic work schedule)		
				Physical work environment (noise at work)		
				Self-reported, measured at baseline		
Harrati et al. 2019 [21]	*N* = 28 843 employees from a major manufacturing firm (18% female); USA	17 years (1996–2013)	American Manufacturing Cohort (AMC)	Physical work environment (air pollution, particulate matter 2.5)	Employment, short-term disability leave (STD), long-term disability leave (LTD), on leave, terminated; registry-based	Trajectories and clusters (cluster analysis, Partitioning Around Medoids); measured in months
				JEM, connected to occupation at baseline		
Hartikainen et al. 2023 [34]	*N* = 9896 receivers of partial SA benefits, aged 45–56 (76.1% female); Finland	7 years (2010–2017)	National administrative registries, 70% random sample of working age population	Biomechanical (physically heavy work)	Work, partial work disability, full work disability, other (unemployment, other type of being outside of labour market); register-based	Trajectories (multiresponse trajectory analysis); measured in months since initial SA spell
				Psychosocial (job control)		
				JEM, connected to occupation at baseline (end of initial SA spell)		
Leinonen et al. 2019 [33]	*N* = 7 180 individuals aged 25–59 attending vocational rehabilitation (62.8% female): Finland	4 years (2 years before and 2 years after rehabilitation); initial rehabilitation between 2008–2010	National administrative registries, 70% random sample of working age population, linked to Finnish Longitudinal Employer-Employee Data (FLEED)	Biomechanical (heavy physical work, kneeling/squatting, repetitive hand movements)	Work, partial work disability, SA, unemployment, DP, other; registry-based	Trajectories (Group-Based Trajectory Modelling); measured in months
				Psychosocial (job strain, monotonous work)		
				JEM, connected to occupation at end of year preceding measurement period of labour market participation		
*Working life patterns based on multiple states*,* cumulative time*						
Chungkham et al. 2024 [29]	*N* = 12 876 individuals aged 50–75 and gainfully employed at baseline, Sweden	2–12 years (multistate model, 2008–2020), 25 years (WLE)	Swedish Longitudinal Occupational Survey of Health (SLOSH), linked to administrative register data	Psychosocial (job strain)	3-state model: working, not in work, death	Multistate model to calculate working life expectancy (WLE) at age 50; measured in years
				Self-reported at baseline	4-state model: working full-time, working part-time, not in work, death	
Lynch et al. 2024 [20]	*N* = 11 540 individuals aged 50–120 years, United Kingdom	8 years (2004/5-2012/3)	English Longitudinal Study of Ageing (ELSA) waves 2–6	Psychosocial (autonomy at work, support at work)	Healthy and working (HWLE), healthy and/or not working, dead; self-reported	Continuous-time multistate model used to calculate Healthy Working Life Expectancy (HWLE)
				Self-reported at baseline for previous month		
Pedersen et al. 2020 [27]	*N* = 1 612 702 individuals aged 30–65 and employed at baseline (49% female); Denmark	4 years (multistate model, 2014–2017), 10–30 years (WLE/WYL)	National administrative registries	Biomechanical (combination variable: sitting, walking/standing, awkward work posture, arms above shoulder height, repetitive arm movements, squatting/kneeling, pushing/pulling, carrying/lifting)	Work, unemployment, SA, temporary exit, DP, death; registry-based	Multistate model to calculate working life expectancy (WLE) and working years lost (WYL) at ages 30, 40, and 50; measured in years
				JEM, connected to occupation at time of transition		
Pedersen et al. 2022 [26]	*N* = 46 169 individuals aged 18–64 and employed at baseline (59% female); Denmark	4 years baseline data collection (2012–2016), 2 years follow-up	Work Environment and Health in Denmark (WEHD) survey, linked to national administrative registries	Biomechanical score (walking/standing, awkward work posture, arms above shoulder height, repetitive arm movements, squatting/kneeling, pushing/pulling, carrying/lifting)	Work, SA, unemployment, temporary exit, DP, retirement, death; registry-based	Expected Labor Market Affiliation (ELMA) Method used to calculate expected length of stay in different states over 2 year period
				Self-reported at baseline, adjusted if individual participated in a new wave (3 survey waves in total)		
Schram et al. 2021 [28]	*N* = 415 105 individuals aged 50–63 and employed at baseline (51% female); Finland	10 years (multistate model, 2004–2014), 13 years (WLE/WYL)	National administrative registries, 70% random sample of working age population	Biomechanical (heavy physical work, kneeling/squatting, heavy lifting, working with hands above shoulder level, awkward posture)	Work, time-restricted work disability, unemployment, economic inactivity, DP, retirement, death; registry-based	Multistate model used to calculate WLE and WYL; measured in years
				JEM connected to occupation in 2004		
Schram et al. 2022 [19]	*N* = 11 800 individuals aged 50–63 years and in work at baseline; The Netherlands	8 years (multistate model, 2010–2018), 16 years (WLE/ WYL)	Dutch Longitudinal Study on Transitions in Employment, Ability and Motivation (STREAM), linked to national administrative registries	Biomechanical (force exertion, static load (standing, posture and kneeling) and vibration)	Work, involuntary exit (disability benefits, unemployment), voluntary exit (economic inactivity, early retirement); registry-based	Multistate model used to calculate WLE and WYL; measured in years
				Psychosocial (psychological job demands, autonomy, emotional demands)		
				Self-reported at baseline		

The studies included several different occupational exposures (see Table 2). Biomechanical exposures were examined in 12 studies [18, 19, 22, 23, 25–28, 30, 31, 33, 34], with a generic measure of physically demanding work as the most common biomechanical exposure. Eleven studies included psychosocial work exposures [18–20, 22, 23, 25, 29, 30, 32–34], predominantly psychosocial job demands, job control or different combinations of work demands and control (including job strain). General physical or chemical work exposures [30], occupational noise [25] and occupational air pollution [21] were included in one study each. Specific occupational exposures were investigated separately in 12 studies, while 5 studies combined several occupational exposures into an overall exposure score. For example, one study combined physical and chemical work exposures (e.g., noise, vibration, heat and cold, dirtiness and smoke) into one grouped variable [30]. Occupational exposures were assessed using job exposure matrices (JEMs), where an exposure level was assigned at job title level, in seven studies [21, 24, 27, 28, 32–34]. Self-reports were used to assess occupational exposures in ten studies [18–20, 22, 23, 25, 26, 29–31]. Fifteen studies reported occupational exposure at one specific time point, while two studies adjusted the assigned biomechanical exposure levels based on new employment information, such as if an individual switched jobs during follow-up [26, 27]. Another study considered cumulative occupational exposure to biomechanical factors based on years exposed [22].

**Table 2:** Studies (*n* = 17) grouped by exposure and outcome

	Outcome			
Exposure category	**Exposure**	**Working life patterns based on work disability**	**Working life patterns based on multiple states**,** trajectories**	**Working life patterns based on multiple states**,** cumulative time**
Biomechanical				
	Physically demanding work (generic)	Hallman et al., 2019; Haukka et al., 2013; Lallukka et al., 2019; Leino-Arjas et al., 2021	Gémes et al., 2023; Leinonen et al., 2019; Hartikainen et al., 2023	Pedersen et al., 2020; Pedersen et al., 2022; Schram et al., 2021; Schram et al., 2022
	Force	Hallman et al., 2019; Lallukka et al., 2019; Shiri et al., 2021		
	Posture	Lallukka et al., 2019; Leino-Arjas et al., 2021; Shiri et al., 2021	Leinonen et al., 2019	
	Repetition	Lallukka et al., 2019	Leinonen et al., 2019	
	Prolonged sitting/standing/walking, keyboard use, using a vibrating tool	Lallukka et al., 2019		
Psychosocial				
	Job demands	Haukka et al., 2013; Leino-Arjas et al., 2021	Gémes et al., 2023	Schram et al., 2022
	Job control	Hallman et al., 2019; Leino-Arjas et al., 2021	Gémes et al., 2023; Hartikainen et al., 2023	Lynch et al., 2024; Schram et al., 2022
	Job demands/control combined	Farrants & Alexanderson, 2022; Salonen et al., 2020; Shiri et al., 2021	Leinonen et al., 2019	Chungkham et al., 2024
	Monotonousness		Gémes et al., 2023; Leinonen et al., 2019	
	Hectic work schedule		Gémes et al., 2023	
	Support at work	Hallman et al., 2019; Haukka et al., 2013		Lynch et al., 2024
Physical & chemical work environment				
	Noise		Gémes et al., 2023	
	Air pollution		Harrati et al., 2019	
	Physical & chemical	Leino-Arjas et al., 2021		

Fourteen of the 17 studies used registry data for outcome measurements, and three studies used self-reported survey data [20, 23, 29]. Seven studies considered only work disability over time, either by identifying sickness absence trajectories [18, 22, 23, 30] or by combining sickness absence and disability pension into trajectories of work disability [24, 32] or the number of working days lost [31] (Table 1). Ten studies included multiple labour market participation states such as paid employment, sickness absence, disability pension, and unemployment. Of these, four characterized working life patterns through labour market participation trajectories [21, 25, 33, 34], four studies estimated WLE and/or WYL [19, 27–29] and one study used a measure of HWLE [20]. One study used a measure of the expected labour market affiliation (ELMA), defined as expected durations spent in paid employment or other labour market participation states during follow-up [26].

For the calculation of labour market participation trajectories, nine studies used a form of trajectory analysis [18, 21–24, 30, 32–34] whilst one study used sequence analysis [25]. Five of the seven studies investigating cumulative time spent in various labour market participation states used multistate models [19, 20, 27–29], whilst one study used the ELMA method [26] and one study calculated the observed number of days [31]. Fourteen studies adjusted for potential confounders (see Supplementary Table S5), mainly sociodemographic factors [18, 20–26, 29–34]. Eleven studies adjusted for health-related variables [18, 20–26, 30, 31, 34]. See Table 3 for a short summary of findings and Supplementary Table S5 for a more detailed overview.

**Table 3:** Summary of associations found in the studies (adjusted models), grouped by exposure and outcome

		Outcome		
Exposure	**Number of studies**	**Working life patterns based on work disability**	**Working life patterns based on multiple states**,** trajectories**	**Working life patterns based on multiple states**,** cumulative time**
*Biomechanical*				
Physically demanding work (generic)	11	++++ increasing SA		++ lower WLE
		++ mixed and high SA		++ more WYL due to work disability and unemployment
				+/- ELMA and WYL, varied by age, gender, and level of exposure
				+ WYL due to involuntary exit
Force	3	++ heavy lifting, increasing SA		
		+ heavy lifting, high SA		
		+ heavy lifting, decreasing SA		
		+ high hand grip force, high SA		
Posture	4	+++ increasing SA	+ resumed work participation after rehabilitation	
		+ intermediate SA		
		+ high SA		
Repetition	2	+ high SA		
Prolonged sitting/standing/walking, keyboard use, using a vibrating tool	1	+ prolonged standing/walking, increasing and high SA		
		- prolonged sitting, keyboard use, increasing and high SA		
*Psychosocial*				
High job demands	3		+ work disability, women	
Low job control	5	+ increasing, mixed and high SA		
Job demands/control combined	5	- high job control regardless of demands, low and high work disability	+ work disability, men	+ job strain, reduced WLE (total, part-time and full-time work)
		+ low job control regardless of demands, stable work disability	+ slightly reduced work	
		- high or medium job demands with high or medium job control, increasing work disability		
		+ job strain, stable and increasing work disability		
		- job strain, higher work disability		
		+ job strain, lower work participation before and after rehabilitation		
Monotonousness	2		+ unemployment and work disability, retirement, men	
Hectic work schedule	1		- unemployment and work disability, men	
High support at work	1			+ higher HWLE
*Physical & chemical work exposures*				
Noise	1		- unemployment and work disability, men	
Air pollution	1		+ work disability, short and long term	
Physical & chemical	1			

SA = sickness absence, (H)WLE = (healthy) working life expectancy, WYL = working years lost, ELMA = expected labour market attachment

+ = study with positive association found, - = study with negative association found. Non-significant associations not shown

### Working life patterns based on sickness absence or disability pension

Seven studies focused solely on work disability using either sickness absence [18, 22, 23, 30] or a combination of sickness absence and disability pension [24, 31, 32] as the outcome. Almost all studies grouped individuals into different trajectories according to their patterns of sickness absence (and disability pension), though one study examined cumulative working days lost [31]. Among the studies based on trajectories, all identified one trajectory with low/no sickness absence/disability pension, which was used as a reference. Other identified trajectories varied slightly between the studies but mainly concerned either increasing levels or consistently high levels of sickness absence/disability pension. Three studies were stratified by gender [24, 30, 31].

Biomechanical factors were included in five studies [18, 22, 23, 30, 31], of which four studies found an association between physically demanding work and trajectories with high levels of sickness absence [18, 22, 23] or work disability [31]. Four studies including pushing, pulling, and handling of heavy loads found an increased probability of sickness absence that was increasing over time or constantly high [22, 23, 30] and a higher number of days lost due to work disability [31]. One study included a measure of cumulative exposure, indicating that both a higher number of biomechanical factors and a longer duration of exposure were associated with a higher risk of increasing or constantly high sickness absence over time [22].

Six studies included psychosocial exposures [18, 23, 24, 30–32]. High psychosocial job demands were not associated with sickness absence trajectories [18, 30]. One of the two studies including job control found an association between low job control and increasing or constantly high sickness absence over time [18], while the other study found no association [30]. Three studies used combination measures of job demands and job control [24, 31, 32]. White collar workers reporting low job control, regardless of the level of job demands, had a higher risk of increasing or constantly high work disability, though the strongest association was among those reporting high job strain [32]. In another study, the strongest associations with membership in the high increasing trajectory were with low job demands/low control for women, and with low demands/high control for men [24]. Another study also found that high job strain was associated with fewer working days lost due to work disability [31].

One study included a composite score of physical and chemical work exposures and found no association with increasing sickness absence trajectories [30].

### Working life patterns based on multiple states: trajectories

All four studies examining trajectories of multiple labour market participation states included employment, sickness absence and disability pension [21, 25, 33, 34], while unemployment was included in three studies [25, 33, 34]. Two studies presented gender-stratified analyses [25, 33].

Three studies included biomechanical and psychosocial factors [25, 33, 34]. Physically heavy work increased the risk of reduced work participation after an episode of part-time sickness absence in one study [34], while another study found no association with reduced work participation before and after vocational rehabilitation [33]. The third study found an association between physically heavy work and a higher risk of periods with work disability among men, but not among women [25]. One study also included kneeling and squatting, reporting an association between high exposure and a trajectory with low work participation prior to vocational rehabilitation and a higher work participation afterwards [33]. The same study also included repetitive hand movements, but an association with reduced work participation was not found.

Concerning psychosocial working conditions, monotonous work was associated with periods of work disability and unemployment in one study [25]. Another study including monotonous work found no association with reduced levels of work participation before and after vocational rehabilitation [33]. Mentally strenuous work was associated with periods of work disability among women, but not among men, in one study [25]. One study including low job control found no association with lower work participation [34]. Job strain was included in one study and increased the risk of negligible work participation before and after vocational rehabilitation [33].

Finally, physical work environment exposures were included in two studies [21, 25]. Noise at work was associated with a lower risk of unemployment and sickness absence/disability pension among men but not among women [25]. Cumulative exposure to work-related total particulate matter was associated with periods of work disability [21].

### Working life patterns based on multiple states: cumulative time

Four of the six studies examining cumulative time spent in multiple labour market participation states used WLE/WYL as outcome measures [19, 27–29], whilst one study focused on the related measure of ELMA, considering the number of days spent in each state [26]. The sixth study used a measure of HWLE where the authors distinguished between time working in good health and a combined state with those out of work and those who were still working but were unhealthy [20].

Four of the six studies included biomechanical factors [19, 26–28]. Employees with high physical work demands had a shorter expected time spent in work compared to those with low physical work demands [19, 26–28]. All six studies found that individuals with high physical work demands lost more working years due to work disability and/or unemployment compared to those with low physical work demands [19, 20, 26–29]. One study found a larger reduction in WLE and an increase in WYL due to disability pension and unemployment at ages 30, 40 and 50 among women with high physical work demands, compared to men [27]. Another study found that women aged 40–49 years old had a larger number of working days lost compared to men with the same level of exposure, while the opposite was found among women and men aged 50–64 years old [26]. A third study (2022) found no gender differences in WLE/WYL at age 50 among employees with high physical work demands [28].

Three studies considered psychosocial exposures [19, 20, 29]. A lack of autonomy at work reduced HWLE [20] and increased time out of work, particularly disability pension and unemployment [19]. Employees with psychological and emotional job demands also had a higher number of WYL [19]. High job strain was associated with lower WLE among both men and women [29].

## Discussion

### Summary of results

To our best knowledge, this scoping review is the first attempt to systematically map the research literature concerning the associations between occupational exposures and dynamic working life patterns. Research on the topic is relatively new, with 16 of the 17 original studies in this scoping review published since 2019. The included studies are heterogeneous, with different occupational exposures and outcomes, study designs, and analytical approaches. Therefore, it was not possible to quantitatively synthesize results.

The majority of the included studies focused on biomechanical factors or psychosocial factors, whilst chemical and physical work environment factors were rarely examined. Individuals with physically demanding work were consistently more likely to have lower levels of work participation and more working time lost to work disability and unemployment, compared to those with less physically demanding work. The associations were most consistent for overall physically demanding work than for specific biomechanical factors. For psychosocial work factors, high job control was associated with patterns of lower work disability and higher HWLE in some studies, though other studies showed no association. Other psychosocial factors showed inconclusive results. Some indications of gender differences were found for biomechanical and psychosocial factors, but associations were inconsistent or inconclusive.

### Considerations for future research

An important point to consider when interpreting the findings is how differences in study designs may have affected the associations and differences observed across the studies. One factor that may contribute to the varying directions and magnitude of associations is the exposure definition and measurement. For example, biomechanical factors were defined and measured in many ways, such as heavy physical work, kneeling and squatting, summary scores, and so forth. Other factors concern the variety of different outcomes and analytical approaches used to capture the dynamics of working life patterns, such as multistate modelling for estimation of WLE and WYL, which are cumulative measures of time spent in different states, compared to the use of trajectory analysis methods which map patterns of transitions over time. As the included studies differ considerably with regards to how exposures, outcomes and methods were defined, measured and applied, comparing and interpreting the results was complicated and applying meta-analytic methods was not possible. To aid future comparisons, a consideration of how definitions and measurements of exposures and outcomes could be harmonised may be beneficial.

A related issue concerns the inclusion of potential confounders and how these should be handled in analyses. The majority of the studies included models that were adjusted for several health-related, sociodemographic, occupational and/or lifestyle variables in addition to the studied occupational exposures. In the six studies that reported both unadjusted and adjusted models, associations were attenuated and no longer significant in adjusted models [23, 30–34]. Moreover, in one study [31], the direction of the association between high job strain and working days lost changed from positive in unadjusted results to negative in adjusted results. Exposures can affect labour market participation directly, or they can have an indirect effect, for example through health issues [35, 36], meaning that health-related variables may lie on a plausible causal pathway between exposure and outcome [37]. Adjusting for these variables would then lead to an underestimation of the total effect of exposures. Considering the role of included variables, and whether these are mediators or effect modifiers rather than confounders, would ensure that studies are measuring the desired effects identified through their chosen study design and covariates. Presenting both unadjusted and adjusted results and being transparent about choices made would aid interpretation of results and comparisons across studies.

The static approach of including only one exposure and/or one labour market participation outcome oversimplifies the complex nature of working life patterns and does not take into account how different exposures and labour market participation states can interact over time. Modelling multiple complex exposures that likely interact with each other, and quantifying their effects on labour market participation, is challenging and calls for repeated measurement over time and advanced statistical approaches. For example, a relatively strong correlation between the occurrence of self-reported biomechanical and psychosocial work factors has been observed, suggesting that these may arise simultaneously from the same working conditions [38, 39]. Only one study included methods to handle intercorrelation of exposures [30], whilst other studies with multiple exposures applied mutual adjustments for the exposures. No studies explored potential interaction effects between occupational exposures. Occupational exposures may also have an immediate effect on labour market participation or may only show (delayed) effects after sustained and accumulated exposure. Including a measure of cumulative exposure or repeated measures of exposure over the working life where possible is therefore recommended for a better understanding of how dynamic exposure(s) at the workplace can affect labour market participation over time. Only one included study calculated a cumulative measure of exposure [22]. Taken together, these considerations indicate a need to model interactions between multiple exposures and include measures of cumulative exposure to better emulate real world conditions.

Only three of the 17 included studies were conducted outside the Nordic countries, and two of these were within Europe. The Nordic countries have unique and comprehensive administrative registries which allow for the inclusion of multiple labour market participation states and creates opportunities to study dynamic working life patterns. Although the Nordic countries show some differences with regards to working conditions and welfare systems, they still share many specific features, which means results are not necessarily generalisable to other, non-Nordic countries. The unobserved effects of policy measures and welfare system design will likely affect the utilisation of welfare benefits such as sickness allowance and should be considered when interpreting and disseminating study results from different countries. Inspiring and stimulating research in other countries with different working conditions and welfare systems, using current methods on other data types, developing and promoting methods that do not require registry data, and exploring opportunities to utilise existing registry or population-level data both within and outside Europe should therefore be a priority.

## Conclusions

The research field of occupational exposures and dynamic working life patterns is small but emerging. This review has identified 17 original studies on the topic. The studies varied in terms of exposures, outcomes, and methods used, making comparisons difficult. However, a consistent result is that heavy physical work was associated with reduced work participation and more time spent on work disability and unemployment. The studies provide valuable insight into the status of the research field and indications how to move forward in terms of study design, in particular how exposures, outcomes, and covariates are defined and operationalised and the shift towards a more dynamic exposure-outcome approach. Future research is warranted to provide valuable insights for employers, policymakers and other stakeholders in developing workplace interventions and policies to prolong and ensure healthy working lives.

## Electronic supplementary material

Below is the link to the electronic supplementary material.

Supplementary Material 1

## Data Availability

No datasets were generated or analysed during the current study.

## Abbreviations

ELMA: Expected labour market affiliation
JEM: Job exposure matrix
(H)WLE: (Healthy) working life expectancy
WYL: Working years lost
