# Supplementary material for: Mapping dynamic working life patterns and the impact of occupational exposures: a scoping review
Source: BMC Public Health. 2025 Jul 3;25:2322. doi: 10.1186/s12889-025-23487-5 (PMC12224796; doi:10.1186/s12889-025-23487-5)
Supplement: Supplementary file 1 — Supplementary Material 1 [file 12889_2025_23487_MOESM1_ESM.docx]

# **Supplementary Material: Mapping dynamic working life patterns and the impact of occupational exposures: a scoping review**

**Table of Contents**

Supplementary Table S1……………………………………………………………………1

Supplementary Table S2……………………………………………………………………2

Supplementary Table S3……………………………………………………………………5

Supplementary Table S4……………………………………………………………………6

Supplementary Table S5……………………………………………………………………7

**Supplementary Table S1. List of occupational exposures and work disability outcomes.**

| **Main exposure category** | **Search terms** |
| --- | --- |
| General | exposure, working conditions, work environment, work risk factor, risk factors at work, work load |
| Psychosocial | psychosocial, job demands, emotional demands, quantitative demands, qualitative demands, decision latitude, decision authority, job control, role conflict, monotonous work, workplace bullying, violence at work, harassment at work |
| Physical, mechanical | bio-mechanic, mechanic, physiologic, ergonomic, physically demanding, physically heavy, repetitive movements, awkward lifting, kneeling, heavy lifting, posture, neck flexion, hands above shoulder, forward bending, vibration |
| Chemical, toxicological, biological | chemical, toxicological, biological factors, neurotoxins, physical agents, radiation, UV, heat, cold, noise, silica, asbestos, benzene, diesel, chromium6, nickel, PAHs, dusts, fibers, solvents, pesticides, metals, metal oxides, nanoparticles, inhalation, electromagnetic fields |
| Work organisation | night work, shift work |
| Working life expectancy/employment-based trajectories | working life expectancy, working years lost, employment-based trajectories, employment-based paths/pathways, employment-based sequences, employment-based clusters (see search terms list for more comprehensive outcome terms) |

**Supplementary Table S2. Search terms per database.**

| **PubMed** | (“exposure”) OR ("work* conditions") OR ("work* environment") OR ("work-related factors") OR (“work risk factor”) OR (“risk factors at work”) OR ("work load") OR ("workload") OR ("psychosocial") OR (“biomechanic*”) OR (“mechanic*“) OR (“physiologic*”) OR ("ergonomic*") OR ("chemical") OR ("toxicological") OR ("biological factors") OR ("partical") OR (“job demands”) OR (“emotional demands”) OR (“quantitative demands”) OR (“qualitative demands”) OR (“decision latitude”) OR (“decision authority”) OR ("autonomy") OR (“job control”) OR (“role conflict”) OR (“monotonous work”) OR (“workplace bullying”) OR (“night work”) OR (“shift work”) OR ("irregular working hours") OR (“violence at work”) OR (“harassment at work”[tiab:~0]) OR ("physical demands") OR (“physically demanding”) OR (“physically heavy”) OR (“repetitive movements”) OR (“awkward lifting”) OR ("kneeling") OR (“heavy lifting”) OR (“posture”) OR (“neck flexion”) OR (“hands above shoulder”[tiab:~0]) OR (“forward bending”) OR (“vibration”) OR ("neurotoxins") OR (“physical agents”) OR (“radiation”) OR (“heat”) OR (“cold”) OR (“noise”) OR ("silica") OR ("asbestos") OR ("benzene") OR ("diesel") OR ("chromium6") OR ("nickel") OR ("PAHs*") OR (“dusts”) OR (“fibers”) OR (“solvents”) OR (“pesticides”) OR (“metals”) OR (“metal oxides”) OR (“nanoparticles”) OR ("electromagnetic fields") OR ("UV") OR ("Occupational Exposure"[Mesh]) OR ("Inhalation Exposure"[Mesh]) OR ("Radiation Exposure"[Mesh])  AND  (“working life expectanc*”) OR (“working life course*”) OR (“employment trajector*”) OR (“employment participation trajectory”[tiab:~0]) OR (“employment participation trajectories”[tiab:~0]) OR (“unemployment trajector*”) OR (“work trajector*”) OR (“work participation trajector*”) OR (“work attachment trajectory”[tiab:~0]) OR (“work attachment trajectories”[tiab:~0]) OR (“working life trajector*”) OR (“labour market trajector*”) OR (“labor market trajector*”) OR (“labour market participation trajectory”[tiab:~0]) OR (“labour market participation trajectories”[tiab:~0]) OR (“labor market participation trajectory”[tiab:~0]) OR (“labor market participation trajectories”[tiab:~0]) OR (“labour market attachment trajector*”) OR (“labor market attachment trajector*”) OR (“labour market attachment*”) OR (“labor market attachment*”) OR (“sickness absence trajector*”) OR (“sick leave trajectory”[tiab:~0]) OR (“sick leave trajectories”[tiab:~0]) OR (“work disability trajector*”) OR (“return to work trajector*”) OR (“trajectories of sick”[tiab:~0]) OR (“trajectories of sickness”[tiab:~0]) OR (“trajectories of work”[tiab:~0]) OR (“trajectories of working”[tiab:~0]) OR (“trajectories of employment” [tiab:~0]) OR (“trajectories of labour” [tiab:~0]) OR (“trajectories of labor”) OR (“trajectories of return to work” [tiab:~0]) OR (“employment path*”) OR (“work path*”) OR (“work participation path”[tiab:~0]) OR (“work participation pathway”[tiab:~0]) OR (“work participation pathways”[tiab:~0]) OR (“working life path”[tiab:~0]) OR (“working life pathway”[tiab:~0]) OR (“working life pathways”[tiab:~0]) OR (“labour market path”[tiab:~0]) OR (“labour market pathway”[tiab:~0]) OR (“labour market pathways”[tiab:~0]) OR (“labor market path”[tiab:~0]) OR (“labor market pathway”[tiab:~0]) OR (“labor market pathways”[tiab:~0]) OR (“labour market participation path”[tiab:~0]) OR (“labour market participation pathway”[tiab:~0]) OR (“labour market participation pathways”[tiab:~0]) OR (“labor market participation path”[tiab:~0]) OR (“labor market participation pathway”[tiab:~0]) OR (“labor market participation pathways”[tiab:~0]) OR (“work disability path"[tiab:~0]) OR (“work disability pathway"[tiab:~0]) OR (“work disability pathways"[tiab:~0]) OR (“paths of labour” [tiab:~0]) OR (“paths of labor” [tiab:~0]) OR (“paths of employment” [tiab:~0]) OR (“paths of work”[tiab:~0]) OR (“paths of working”[tiab:~0]) OR (“pathways of labour” [tiab:~0]) OR (“pathways of labor” [tiab:~0]) OR (“pathways of employment” [tiab:~0]) OR (“pathways of work”[tiab:~0]) OR (“pathways of working”[tiab:~0]) OR (“working years lost”) OR (“retirement expectancy”[tiab:~0]) OR (“retirement expectancies”[tiab:~0]) OR (“unemployment expectancy”[tiab:~0]) OR (“unemployment expectancies”[tiab:~0]) OR (“labour market expectancy”[tiab:~0]) OR (“labour market expectancies”[tiab:~0]) OR (“labor market expectancy”[tiab:~0]) OR (“labor market expectancies”[tiab:~0]) OR (“employment expectancy”[tiab:~0]) OR (“employment expectancies”[tiab:~0]) OR (“labour market affiliation”) OR (“labor market affiliation”) OR (“work sequence*”) OR (“employment sequence”[tiab:~0]) OR (“employment sequences”[tiab:~0]) OR (“labour market sequence”[tiab:~0]) OR (“labour market sequences”[tiab:~0]) OR (“labor market sequence”[tiab:~0]) OR (“labor market sequences”[tiab:~0]) OR (“employment pattern*”) OR (“labour market pattern*”) OR (“labor market pattern*”) OR (“labour market participation pattern”[tiab:~0]) OR (“labour market participation patterns”[tiab:~0]) OR (“labor market participation pattern”[tiab:~0]) OR (“labor market participation patterns”[tiab:~0]) OR (“work pattern*”) OR (“work participation pattern”[tiab:~0]) OR (“work participation patterns”[tiab:~0]) OR (“working life pattern”[tiab:~0]) OR (“working life patterns”[tiab:~0]) OR (“patterns of labour” [tiab:~0]) OR (“patterns of labor” [tiab:~0]) OR (“patterns of employment” [tiab:~0]) OR (“patterns of work” [tiab:~0]) OR (“patterns of working” [tiab:~0]) OR (“employment cluster*”) OR (“work cluster*”) OR (“work participation cluster”[tiab:~0]) OR (“work participation clusters”[tiab:~0]) OR (“work participation clustering”[tiab:~0]) OR (“labour market cluster”[tiab:~0]) OR (“labour market clusters”[tiab:~0]) OR (“labour market clustering”[tiab:~0]) OR (“labor market cluster” [tiab:~0]) OR (“labor market clusters”[tiab:~0]) OR (“labor market clustering”[tiab:~0]) OR (“labour market participation cluster” [tiab:~0]) OR (“labour market participation clusters” [tiab:~0]) OR (“labour market participation clustering” [tiab:~0]) OR (“labor market participation cluster” [tiab:~0]) OR (“labor market participation clusters” [tiab:~0]) OR (“labor market participation clustering” [tiab:~0]) OR (“clusters of work” [tiab:~0]) OR (“clusters of working” [tiab:~0]) OR (“clusters of employment” [tiab:~0]) OR (“clusters of labour” [tiab:~0]) OR (“clusters of labor” [tiab:~0]) OR (“employment profile*”) OR (“work profile*”) OR (“labour market profile” [tiab:~0]) OR (“labour market profiles” [tiab:~0]) OR (“labor market profile” [tiab:~0]) OR (“labor market profiles” [tiab:~0]) OR (“labour market participation profile” [tiab:~0]) OR (“labour market participation profiles” [tiab:~0]) OR (“labor market participation profile” [tiab:~0]) OR (“labor market participation profiles” [tiab:~0]) OR (“profiles of work” [tiab:~0]) OR (“profiles of working” [tiab:~0]) OR (“profiles of employment” [tiab:~0]) OR (“profiles of labour” [tiab:~0]) OR (“profiles of labor” [tiab:~0])  **Filters:** human |
| --- | --- |
| **Embase** | exposure* OR work* conditions OR work* environment OR work-related factors OR work risk factor OR risk factors at work OR work load OR workload OR psychosocial OR biomechanic* OR mechanic* OR physiologic* OR ergonomic* OR chemical OR toxicological OR biological factors OR partical OR job demands OR emotional demands OR quantitative demands OR qualitative demands OR decision latitude OR decision authority OR autonomy OR job control OR role conflict OR monotonous work OR workplace bullying OR night work OR shift work OR irregular working hours OR violence at work OR harassment at work OR physical demands OR physically demanding OR physically heavy OR repetitive movements OR awkward lifting OR kneeling OR heavy lifting OR posture OR neck flexion OR hands above shoulder OR forward bending OR vibration OR neurotoxins OR physical agents OR radiation OR heat OR cold OR noise OR silica OR asbestos OR benzene OR diesel OR chromium6 OR nickel OR PAHs* OR dusts OR fibers OR solvents OR pesticides OR metals OR metal oxides OR nanoparticles OR electromagnetic fields OR UV OR Occupational Exposure/ OR Inhalation Exposure/ OR Radiation Exposure/  AND  working life expectanc* OR working life course* OR employment trajector* OR employment participation trajector* OR unemployment trajector* OR work trajector* OR work participation trajector* OR work attachment trajector* OR working life trajector* OR labour market trajector* OR labour market participation trajector* OR labour market attachment trajector* OR labor market trajector* OR labor market participation trajector* OR labor market attachment trajector* OR sickness absence trajector* OR sick leave trajector* OR work disability trajector* OR return to work trajector* OR trajectories of sick* OR trajectories of work* OR trajectories of employment OR trajectories of labour OR trajectories of labor OR trajectories of return to work OR employment path* OR work path* OR work participation path* OR working life path* OR labour market path* OR labour market participation path* OR labor market path* OR labor market participation path* OR work disability path* OR paths of labour OR paths of labor OR paths of employment OR paths of work* OR pathways of labour OR pathways of labor OR pathways of employment OR pathways of work* OR working years lost OR retirement expectanc* OR unemployment expectanc* OR labour market expectanc* OR labor market expectanc* OR employment expectanc* OR labour market affiliation OR labor market affiliation OR work sequence* OR employment sequence* OR labour market sequence* OR labor market sequence* OR employment pattern* OR labour market pattern* OR labour market participation pattern* OR labor market pattern* OR labor market participation pattern* OR work pattern* OR work participation pattern* OR working life pattern* OR patterns of labour OR patterns of labor OR patterns of employment OR patterns of work* OR employment cluster* OR work cluster* OR work participation cluster* OR labour market cluster* OR labour market participation cluster* OR labor market cluster* OR labor market participation cluster* OR clusters of work* OR clusters of employment OR clusters of labour OR clusters of labor OR employment profile* OR work profile* OR labour market profile* OR labour market participation profile* OR labor market profile* OR labor market participation profile* OR profiles of work* OR profiles of employment OR profiles of labour OR profiles of labor  **Filters:** human |
| **Web of Science** | (“exposure”) OR ("work* conditions") OR ("work* environment") OR ("work-related factors") OR (“work risk factor”) OR (“risk factors at work”) OR ("work load") OR ("workload") OR ("psychosocial") OR (“biomechanic*”) OR (“mechanic*“) OR (“physiologic*”) OR ("ergonomic*") OR ("chemical") OR ("toxicological") OR ("biological factors") OR ("partical") OR (“job demands”) OR (“emotional demands”) OR (“quantitative demands”) OR (“qualitative demands”) OR (“decision latitude”) OR (“decision authority”) OR ("autonomy") OR (“job control”) OR (“role conflict”) OR (“monotonous work”) OR (“workplace bullying”) OR (“night work”) OR (“shift work”) OR ("irregular working hours") OR (“violence at work”) OR (“harassment at work”) OR ("physical demands") OR (“physically demanding”) OR (“physically heavy”) OR (“repetitive movements”) OR (“awkward lifting”) OR ("kneeling") OR (“heavy lifting”) OR (“posture”) OR (“neck flexion”) OR (“hands above shoulder”) OR (“forward bending”) OR (“vibration”) OR ("neurotoxins") OR (“physical agents”) OR (“radiation”) OR (“heat”) OR (“cold”) OR (“noise”) OR ("silica") OR ("asbestos") OR ("benzene") OR ("diesel") OR ("chromium6") OR ("nickel") OR ("PAHs*") OR (“dusts”) OR (“fibers”) OR (“solvents”) OR (“pesticides”) OR (“metals”) OR (“metal oxides”) OR (“nanoparticles”) OR ("electromagnetic fields") OR ("UV") OR ("Occupational Exposure") OR ("Inhalation Exposure") OR ("Radiation Exposure")  AND  (“working life expectanc*”) OR (“working life course*”) OR (“employment trajector*”) OR (“employment participation trajector*”) OR (“unemployment trajector*”) OR (“work trajector*”) OR (“work participation trajector*”) OR (“work attachment trajector*”) OR (“working life trajector*”) OR (“labour market trajector*”) OR (“labour market participation trajector*”) OR (“labour market attachment trajector*”) OR (“labor market trajector*”) OR (“labor market participation trajector*”) OR (“labor market attachment trajector*”) OR (“sickness absence trajector*”) OR (“sick leave trajector*”) OR (“work disability trajector*”) OR (“return to work trajector*”) OR (“trajectories of sick*”) OR (“trajectories of work*”) OR (“trajectories of employment”) OR (“trajectories of labour”) OR (“trajectories of labor”) OR (“trajectories of return to work”) OR (“employment path*”) OR (“work path*”) OR (“work participation path*”) OR (“working life path*”) OR (“labour market path*”) OR (“labour market participation path*”) OR (“labor market path*”) OR (“labor market participation path*”) OR (“work disability path*") OR (“paths of labour”) OR (“paths of labor”) OR (“paths of employment”) OR (“paths of work*”) OR (“pathways of labour”) OR (“pathways of labor”) OR (“pathways of employment”) OR (“pathways of work*”) OR (“working years lost”) OR (“retirement expectanc*”) OR (“unemployment expectanc*”) OR (“labour market expectanc*”) OR (“labor market expectanc*”) OR (“employment expectanc*”) OR (“labour market affiliation”) OR (“labor market affiliation”) OR (“work sequence*”) OR (“employment sequence*”) OR (“labour market sequence*”) OR (“labor market sequence*”) OR (“employment pattern*”) OR (“labour market pattern*”) OR (“labour market participation pattern*”) OR (“labor market pattern*”) OR (“labor market participation pattern*”) OR (“work pattern*”) OR (“work participation pattern*”) OR (“working life pattern*”) OR (“patterns of labour”) OR (“patterns of labor”) OR (“patterns of employment”) OR (“patterns of work*”) OR (“employment cluster*”) OR (“work cluster*”) OR (“work participation cluster*”) OR (“labour market cluster*”) OR (“labour market participation cluster*”) OR (“labor market cluster*”) OR (“labor market participation cluster*”) OR (“clusters of work*”) OR (“clusters of employment”) OR (“clusters of labour”) OR (“clusters of labor”) OR (“employment profile*”) OR (“work profile*”) OR (“labour market profile*”) OR (“labor market profile*”) OR (“labour market participation profile*”) OR (“labor market participation profile*”) OR (“profiles of work*”) OR (“profiles of employment”) OR (“profiles of labour”) OR (“profiles of labor”)  **Filters:** article |
| **Scopus** | TITLE-ABS-KEY(({exposure} OR {work* conditions} OR {work* environment} OR {work-related factors} OR {work risk factor} OR {risk factors at work} OR {work load} OR {workload} OR {psychosocial} OR {biomechanic*} OR {mechanic*} OR {physiologic*} OR {ergonomic*} OR {chemical} OR {toxicological} OR {biological factors} OR {partical} OR {job demands} OR {emotional demands} OR {quantitative demands} OR {qualitative demands} OR {decision latitude} OR {decision authority} OR {autonomy} OR {job control} OR {role conflict} OR {monotonous work} OR {workplace bullying} OR {night work} OR {shift work} OR {irregular working hours} OR {violence at work} OR {harassment at work} OR {physical demands} OR {physically demanding} OR {physically heavy} OR {repetitive movements} OR {awkward lifting} OR {kneeling} OR {heavy lifting} OR {posture} OR {neck flexion} OR {hands above shoulder} OR {forward bending} OR {vibration} OR {neurotoxins} OR {physical agents} OR {radiation} OR {heat} OR {cold} OR {noise} OR {silica} OR {asbestos} OR {benzene} OR {diesel} OR {chromium6} OR {nickel} OR {PAHs*} OR {dusts} OR {fibers} OR {solvents} OR {pesticides} OR {metals} OR {metal oxides} OR {nanoparticles} OR {electromagnetic fields} OR {UV} OR {Occupational Exposure} OR {Inhalation Exposure} OR {Radiation Exposure})  AND  ({working life expectanc*} OR {working life course*} OR {employment trajector*} OR {employment participation trajector*} OR {unemployment trajector*} OR {work trajector*} OR {work participation trajector*} OR {work attachment trajector*} OR {working life trajector*} OR {labour market trajector*} OR {labour market participation trajector*} OR {labour market attachment trajector*} OR {labor market trajector*} OR {labor market participation trajector*} OR {labor market attachment trajector*} OR {sickness absence trajector*} OR {sick leave trajector*} OR {work disability trajector*} OR {return to work trajector*} OR {trajectories of sick*} OR {trajectories of work*} OR {trajectories of employment} OR {trajectories of labour} OR {trajectories of labor} OR {trajectories of return to work} OR {employment path*} OR {work path*} OR {work participation path*} OR {working life path*} OR {labour market path*} OR {labour market participation path*} OR {labor market path*} OR {labor market participation path*} OR {work disability path*} OR {paths of labour} OR {paths of labor} OR {paths of employment} OR {paths of work*} OR {pathways of labour} OR {pathways of labor} OR {pathways of employment} OR {pathways of work*} OR {working years lost} OR {retirement expectanc*} OR {unemployment expectanc*} OR {labour market expectanc*} OR {labor market expectanc*} OR {employment expectanc*} OR {labour market affiliation} OR {labor market affiliation} OR {work sequence*} OR {employment sequence*} OR {labour market sequence*} OR {labor market sequence*} OR {employment pattern*} OR {labour market pattern*} OR {labour market participation pattern*} OR {labor market pattern*} OR {labor market participation pattern*} OR {work pattern*} OR {work participation pattern*} OR {working life pattern*} OR {patterns of labour} OR {patterns of labor} OR {patterns of employment} OR {patterns of work*} OR {employment cluster*} OR {work cluster*} OR {work participation cluster*} OR {labour market cluster*} OR {labour market participation cluster*} OR {labor market cluster*} OR {labor market participation cluster*} OR {clusters of work*} OR {clusters of employment} OR {clusters of labour} OR {clusters of labor} OR {employment profile*} OR {work profile*} OR {labour market profile*} OR {labor market profile*} OR {labour market participation profile*} OR {labor market participation profile*} OR {profiles of work*} OR {profiles of employment} OR {profiles of labour} OR {profiles of labor}))  **Filters:** TITLE-ABS-KEY (title, abstract, keywords) |

**Supplementary Table S3. Screening guidelines, agreed prior to search.**

| **PUBLICATION YEAR** | |
| --- | --- |
| **INCLUDE** | **EXCLUDE** |
| All years |  |

| **LANGUAGE** | |
| --- | --- |
| **INCLUDE** | **EXCLUDE** |
| All European languages |  |

| **PUBLICATION TYPE** – Is this the right publication type? | |
| --- | --- |
| **INCLUDE** | **EXCLUDE** |
| Peer-reviewed primary/original article | Review article, meta-analysis, book, book chapter, editorial, working paper, report etc. |

| **MAIN VARIABLES** – Does the study have both exposure and work outcome variables? | |
| --- | --- |
| **INCLUDE** | **EXCLUDE** |
| Any kind of occupational exposure (full list described in idea paper)  Employment-based trajectories, working life expectancy | Social support, job stress, no exposure information  Work status measured only at one time point |

| **POPULATION**: | |
| --- | --- |
| **INCLUDE** | **EXCLUDE** |
| Working-age population, sector-, industry-, or occupation-specific population | Articles focusing on children, adolescents, individuals well over retirement age (e.g., 85+) |

Based on a template from:

Screening for studies in systematic, scoping, and other knowledge syntheses: strategies for improvement.

Prepared by, Ayala, AP. Last modified April 27, 2020.

Gerstein Science Information Centre

https://osf.io/p92h4/

**Supplementary Table S4. Data extraction template, created in Covidence.**

| **Heading** | **Description** |
| --- | --- |
| Covidence # | Reference number for the article, assigned by Covidence |
| Study ID | ID generated by Covidence |
| Reviewer name | Name of the author who reviewed/extracted data |
| Study authors | Authors of the article |
| Study title | Study title |
| Year published | Year article was published |
| Country in which the study was published | Multiple choice: Denmark, Sweden, Finland, Netherlands, United States, Other |
| Aim/research question(s) | Clearly state aim and any research questions |
| Study design | Multiple choice: Randomised controlled trial, Non-randomised experimental study, Cohort study, Cross-sectional study, Case control study, Other |
| Type of data | Multiple choice (as many as apply): Registry data, Survey data, JEM (job exposure matrix), Other |
| If specific cohorts/surveys are used, specify name(s) of these here (e.g., Survey of Living Conditions) | Write in name of specific cohorts/surveys |
| Period of exposure measurement | Can use either exact dates or year |
| Period of outcome measurement | Can use either exact dates or year |
| Occupational exposure(s) | Be as specific as possible, write down all occupational exposures investigated |
| Work participation outcome(s) | Outcomes included in the study, e.g., employment, unemployment, sickness absence |
| Work participation measurement(s) | How work participation is measured, e.g., working life expectancy, working years lost, trajectories |
| Unit of measurement for outcome | Days, months, years etc. |
| Type of population | Working-age population (including those not employed), employed individuals, patient group, occupational group, etc. |
| Age range of study population |  |
| Gender | % females, if both genders are included or only one |
| Total number of participants |  |
| Summary of statistical methods | Outline methods used, specific analysis methods (e.g., cluster analysis) |
| Covariates in the analysis |  |
| Positive, no, or negative association? | Please give the overall result of the association between the exposure(s) measured and the work participation outcome |
| Limitations of the study | Limitations as identified by the authors of the study |
| Snowball – potential papers identified from references | If you see any papers in the reference list that could be relevant, add them here so they can be screened |
| Additional notes |  |

**Supplementary Table S5. Comprehensive summary of results from the included studies (N=17).**
SA: sickness absence, DP: disability pension, OR: odds ratio, IRR: incidence rate ratio, RRR: relative risk ratio, (H)WLE: (healthy) working life expectancy, ELMA: expected labour market attachment, CI: confidence interval, NS: not statistically significant, S: statistically significant

| Outcome | Author, year | Exposure measures/ categories | Confounders | Results | Comments |
| --- | --- | --- | --- | --- | --- |
| *Working life patterns based on work disability* | | |  |  |  |
| SA trajectories | Hallman et al. 2019 | Biomechanical: perceived physical exertion (scale); lifting and carrying (scale); pushing and pulling (scale)  Psychosocial: decision authority (scale) | Personal factors:  age  Occupational factors: occupational class, seniority in the job (years)  Lifestyle factors:  vigorous leisure-time physical activity, smoking, alcohol intake, BMI  Health-related factors: intensity, duration, and localization of pain in seven body sites (neck/shoulders, elbows, hands/wrists, lower back, hips, knees, and feet/ankles); pain interference in physical and social activities | Four trajectories identified: no sick leave (76%), Few days - increasing trajectory (FdI, 19%), Some days - decreasing trajectory (SdD, 3%), Some days - increasing trajectory (SdI, 2%). No sick leave was used as the reference group.  Adjusted results (OR (95% CI)):  FdI trajectory:  physical exertion at work: 1.23 (1.13, 1.35)  pushing/pulling: 1.15 (1.01, 1.30)  lifting and carrying: 1.06 (0.94, 1.20)  decision authority: 1.00 (0.99, 1.01)  SdD trajectory:  physical exertion at work: 1.19 (0.98, 1.48)  pushing/pulling: 1.32 (1.00, 1.73)  lifting and carrying: 0.91 (0.68, 1.21)  decision authority: 1.01 (0.98, 1.03)  SdI trajectory:  physical exertion at work: 1.30 (1.01, 1.72)  pushing/pulling: 1.89 (1.33, 2.79)  lifting and carrying: 1.73 (1.24, 2.48)  decision authority: 0.98 (0.96, 1.00) | No unadjusted results reported. |
| SA trajectories | Haukka et al. 2013 | Biomechanical:  heavy physical work (involving lifting and carrying) (dichotomized)  Psychosocial:  job demands; job control (dichotomized) | Personal factors:  Age, education  Occupational factors:  Social support at work  Lifestyle factors:  leisure-time physical activity, BMI, smoking, sleep disorders  Health-related factors:  number of pain sites, chronic diseases (musculoskeletal, mental, cardiovascular, and other somatic diseases as separate variables) | Four trajectories identified: low (59%), high (9%), ascending (21%) and mixed (11%). The low trajectory group was used as a reference group.  Adjusted results (OR (95% CI)):  Ascending  heavy physical work: 1.23 (1.01, 1.51)  high job demands: 0.89 (0.73, 1.08)  low job control: 1.37 (1.12, 1.66)  Mixed  heavy physical work: 1.35 (1.04, 1.75)  high job demands: 1.16 (0.91, 1.48)  low job control: 1.47 (1.15, 1.89)  High  heavy physical work: 1.95 (1.48, 2.58)  high job demands: 1.05 (0.80, 1.37)  low job control: 1.65 (1.25, 2.18) | Main association studied in paper was between musculoskeletal pain and sickness absence trajectories; occupational exposures were part of model adjustment and only present in fully adjusted model. |
| SA trajectories | Lallukka et al. 2019 | Biomechanical (years):  strenuous physical work; handling of loads >20kg; handling of loads >5kg; manual squatting or kneeling; working in bent postures; working with the arms above shoulder level; work requiring high handgrip force; repetitive arm movement; prolonged standing or walking; prolonged sitting; prolonged keyboard use; use of a vibrating tool; frequent manual work  Summary variables:  (1) factors that could decrease the risk of SA (sitting and computer work combined) and (2) number of factors that could increase the risk of SA, i.e., exposure to the nine work-related risk factors, further classified into four groups: 0, 1, 2–3 or 4 or more work-related exposures. | Personal factors:  Age, gender, education, marital status  Occupational factors:  job strain (high job demands/low job control), social support at work    Lifestyle factors:  BMI, current daily smoking, alcohol dependence, leisure time physical activity, sleep problems  Health-related factors:  musculoskeletal disorders, chronic diseases (cardiovascular-, respiratory-  or neurological disease, diabetes, cancer, peptic ulcer or permanent injury), mental disorders | Three SA trajectories identified: consistent low (54.6%), slowly increasing (33.7%) and high (11.7%). Consistent low was used as the reference group.  Adjusted results (OR (95% CI)):  Slowly increasing SA trajectory  strenuous physical work overall: 1.40 (1.14, 1.73) (exposed 1-15 yrs), 1.26 (1.01, 1.59) (exposed >15 yrs)  handling of loads >20kg: 1.53 (1.18, 1.97) (1-15 yrs), 1.36 (1.03, 1.80) (>15 yrs)  handling of loads >5kg: 1.35 (1.03, 1.76) (1-15 yrs), 1.14 (0.84, 1.55) (>15 yrs)  work that requires high hand  grip force: 1.37 (1.08, 1.73) (1-15 yrs), 1.27 (1.00, 1.63) (>15 yrs)  Arms above shoulder level: 1.22 (0.96, 1.55) (1-15 yrs), 0.99 (0.76, 1.28) (>15 yrs)  bent postures: 1.15 (0.94, 1.41) (1-15 yrs), 1.18 (0.94, 1.48) (>15 yrs)  squatting or kneeling: 1.46 (1.16, 1.82) (1-15 yrs), 1.39 (1.09, 1.78) (>15 yrs)  repetitive arm movement: 1.02 (0.86, 1.22) (1-15 yrs), 1.18 (0.96, 1.44) (>15 yrs)  prolonged standing or walking: 1.42 (1.19, 1.68) (1-15 yrs), 1.42 (1.16, 1.73) (>15 yrs)  using a vibrating tool: 0.97 (0.64, 1.48) (1-15 yrs), 0.93 (0.62, 1.40) (>15 yrs)  Number of risk factors experienced simultaneously: 1.23 (1.02, 1.48) (1 exposure), 1.24 (1.01, 1.52) (2-3), 1.54 (1.25, 1.89) (≥4)  prolonged sitting: 0.66 (0.55, 0.80) (1-15 yrs), 0.86 (0.69, 1.07) (>15 yrs)  keyboard use: 0.79 (0.64, 0.95) (1-15 yrs), 0.91 (0.71, 1.17) (>15 yrs)  either prolonged sitting or keyboard use: 0.79 (0.65, 0.95)  prolonged sitting and keyboard use combined: 0.73 (0.63, 0.87)  High SA trajectory  strenuous physical work overall: 1.85 (1.37, 2.49) (1-15 yrs), 1.82 (1.32, 2.51) (>15 yrs)  handling of loads >20kg: 1.73 (1.19, 2.51) (1-15 yrs), 1.65 (1.11, 2.44) (>15 yrs)  handling of loads >5kg: 1.86 (1.28, 2.69) (1-15 yrs), 2.05 (1.38, 3.03) (>15 yrs)  work that requires high hand  grip force: 1.69 (1.20, 2.39) (1-15 yrs), 1.82 (1.30, 2.54) (>15 yrs)  arms above shoulder level: 1.45 (1.03, 2.05) (1-15 yrs), 1.26 (0.88, 1.81) (>15 yrs)  bent postures - 1.70 (1.29, 2.27) (1-15 yrs), 1.70 (1.25, 2.32) (>15 yrs)  squatting or kneeling: 1.71 (1.23, 2.37) (1-15 yrs), 1.72 (1.22, 2.44) (>15 yrs)  repetitive arm movement: 1.26 (0.96, 1.64) (1-15 yrs), 1.87 (1.41, 2.48) (>15 yrs)  prolonged standing or walking: 1.87 (1.44, 2.42) (1-15 yrs), 2.09 (1.58, 2.78) (>15 yrs)  using a vibrating tool: 1.50 (0.83, 2.69) (1-15 yrs), 2.06 (1.23, 3.45) (>15 yrs)  Number of risk factors experienced simultaneously: 1.39 (1.02, 1.90) (1 exposure), 1.84 (1.34, 2.53) (2-3), 2.71 (1.99, 3.69) (≥4)  prolonged sitting: 0.48 (0.36, 0.66) (1-15 yrs), 0.86 (0.62, 1.18) (>15 yrs)  keyboard use: 0.55 (0.40, 0.77) (1-15 yrs), 0.81 (0.56, 1.18) (>15 yrs)  either prolonged sitting or keyboard use: 0.66 (0.49, 0.88)  prolonged sitting and keyboard use combined: 0.57 (0.43, 0.77). | No unadjusted results reported. |
| SA trajectories | Leino-Arjas et al. 2021 | Biomechanical: awkward work postures (summary score of bent or twisted postures, otherwise poor posture, repetitive movements), physical demands (summary score of carrying and standing, frequent movement)  Psychosocial (score):  job control (score); mental demands.  Physical and chemical work exposures (summary score): dirtiness, dust, smoke, steam etc.; risk of accident; noise; vibration; lighting and glare; heat, cold, and changing temperature; dryness; restless environment; noisy people  All exposure scores categorized as low, moderate and high. | Personal factors:  age, education, socioeconomic status, family income  Occupational factors: satisfaction with management  Lifestyle factors:  smoking, BMI, leisure time physical activity, sleep problems  Health-related factors:  injuries and diseases or disorders (musculoskeletal cardiovascular, respiratory, mental, other somatic disorders (neurological or sensory, gastro-intestinal, genitourinary, skin, neoplasms, hormonal and metabolic, and any diseases of the blood, infectious diseases, and congenital anomalies) | Three SA trajectories: consistent low (67% of men, 72% of women), consistent intermediate (23% of men, 21% of women), and increasing (10% of men, 7% of women). Consistent low was used as reference group.  Adjusted results (OR (95% CI)):  Consistent intermediate, men  physical and chemical exposures: 1.20 (0.86, 1.66) (moderate exposure), 1.32 (0.92, 1.88) (high exposure)  physical demands: 0.97 (0.71, 1.32) (moderate), 1.07 (0.78, 1.47) (high)  awkward work postures: 1.80 (1.26, 2.57) (moderate), 1.92 (1.29, 2.86) (high)  job control: 0.79 (0.60, 1.06) (moderate), 0.84 (0.61, 1.17) (high)  mental demands: 0.91 (0.69, 1.21) (moderate), 1.03 (0.78, 1.36) (high)  Increasing, men  physical and chemical exposures: 1.70 (0.96, 3.04) (moderate), 1.22 (0.63, 2.36) (high)  physical demands: 1.37 (0.77, 2.42) (moderate), 1.62 (0.90, 2.91) (high)  awkward work postures: 0.81 (0.44, 1.52) (moderate), 1.25 (0.65, 2.41) (high)  job control: 0.82 (0.50, 1.36) (moderate), 0.92 (0.51, 1.65) (high)  mental demands: 1.19 (0.76, 1.88) (moderate), 0.60 (0.34, 1.03) (high)  Consistent intermediate, women  physical and chemical exposures: 1.09 (0.83, 1.44) (moderate), 1.04 (0.77, 1.39) (high)  physical demands: 1.29 (0.94, 1.77) (moderate), 1.41 (0.98, 2.02) (high)  awkward work postures: 1.35 (0.98, 1.86) (moderate), 1.55 (1.10, 2.12) (high)  job control: 0.83 (0.63, 1.10) (moderate), 0.87 (0.65, 1.17) (high)  mental demands: 0.81 (0.61, 1.07) (moderate), 0.63 (0.46, 0.88) (high)  Increasing, women  physical and chemical exposures: 1.03 (0.57, 1.97) (moderate), 0.78 (0.39, 1.58) (high)  physical demands: 0.80 (0.37, 1.73) (moderate), 1.22 (0.53, 2.80) (high)  awkward work postures: 1.28 (0.57, 2.87) (moderate), 1.45 (0.63, 3.35) (high)  job control: 0.53 (0.26, 1.11) (moderate), 1.02 (0.52, 2.04) (high)  mental demands: 0.96 (0.45, 1.65) (moderate), 0.62 (0.28, 1.40) (high) | Gender stratified analyses.  Associations were tested in two models: (1) adjusted for age only and (2) mutually adjusted for all exposures and all potential confounders, including health-related variables.  In the base model (age-adjusted) the following associations were statistically significant:  physical and chemical exposures with intermediate trajectory (both moderate and high, both genders) and with increasing trajectory (both moderate and high, men only)  physical work demands with intermediate trajectory (both moderate and high, both genders) and with increasing trajectory (only high, both genders)  awkward work postures with intermediate trajectory (both moderate and high, both genders) and with increasing trajectory (only high, both genders)  job control with intermediate trajectory (both moderate and high, both genders) and with increasing trajectory (only high, both genders)  mental demand with intermediate trajectory (both moderate and high, women only) and with high trajectory (only high, both genders).  Associations were negative (reduced risk of membership in trajectories) for both job control and mental demands. |
| SA/DP trajectories | Farrants & Alexanderson 2022 | Job demands & job control combinations (9 categories) | Personal factors:  age, sex, type of living area, education, country of birth, family situation (civil status and children)  Occupational factors:  changed branch of industry, changed sector, changed occupation, size of workplace | Four trajectories identified: No SA/DP (73%), low SA/DP (24%), High SA/DP (2%), and increasing SA/DP (1%). No SA/DP used as reference.  Adjusted results (OR (95% CI)):  Low SA/DP trajectory  High demand/high control: 0.90 (0.85, 0.94)  High demand/medium control: 0.98 (0.94, 1.03)  High demand/low control: 1.01 (0.97, 1.06)  Medium demand/high control: 0.92 (0.87, 0.96)  Medium demand/low control: 1.03 (0.98, 1.08)  Low demand/high control: 0.92 (0.87, 0.97)  Low demand/medium control: 0.99 (0.94, 1.04)  Low demand/low control: 1.22 (0.97, 1.07)  Increasing SA/DP trajectory  High demand/high control: 0.89 (0.71, 1.13)  High demand/medium control: 1.17 (0.96, 1.42)  High demand/low control: 1.19 (0.98, 1.44)  Medium demand/high control: 0.97 (0.77, 1.21)  Medium demand/low control: 1.11 (0.90, 1.37)  Low demand/high control: 0.96 (0.76, 1.21)  Low demand/medium control: 1.05 (0.85, 1.30)  Low demand/low control: 1.06 (0.86, 1.30)  High SA/DP trajectory  High demand/high control: 0.51 (0.39, 0.66)  High demand/medium control: 1.00 (0.82, 1.21)  High demand/low control: 1.35 (1.13, 1.62)  Medium demand/high control: 0.61 (0.48, 0.77)  Medium demand/low control: 1.47 (1.21, 1.78)  Low demand/high control: 0.66 (0.52, 0.84)  Low demand/medium control: 0.85 (0.69, 1.06)  Low demand/low control: 1.51 (1.25, 1.83) | Associations were tested in two models: (1) unadjusted and (2) mutually adjusted for variables in the model.  In the crude model the following associations were statistically significant:  High demand/high control- all trajectories (reduced risk)  High demand/medium control-all trajectories (increased risk)  High demand/low control- all trajectories (increased risk)  Medium demand/high control- all trajectories (reduced risk)  Medium demand/low control-all trajectories (increased risk)  Low demand/high control- all trajectories (reduced risk)  Low demand/medium control-low SA/DP (increased risk) and high SA/DP (reduced risk)  Low demand/low control- all trajectories (increased risk).  The highest risk was for high demand/low control and high SA/DP trajectory.  The lowest risk was for medium demand/high control and high SA/DP trajectory. |
| SA/DP trajectories | Salonen et al. 2020 | Job demands and job control combinations (9 categories) | Personal factors:  age, country of birth, education, type of living area, family composition  Health-related factors:  previous work disability benefits (having part-time SA, full-time and part-time DP) | Three trajectories were identified for women: low stable (72%), medium stable (20%), and high increasing (8%). Two trajectories were identified for men: low stable (91%) and high increasing (9%). Low stable was used as reference group for both genders.  Adjusted results (OR (95% CI)):  Medium stable SA/DP trajectory, women  High demand/high control: 1.0 (NS)  High demand/medium control: 0.98 (NS)  High demand/low control: 1.30 (S)  Medium demand/high control: 1.02 (NS)  Medium demand/medium control: 1.00 (NS)  Medium demand/low control: 1.08 (S)  Low demand/high control: 1.0 (NS)  Low demand/medium control: 1.19 (S)  Low demand/low control: 1.33 (S)  High increasing SA/DP trajectory, women  High demand/high control: 0.79 (S)  High demand/medium control: 0.83 (S)  High demand/low control: 1.17 (S)  Medium demand/high control: 0.85 (S)  Medium demand/medium control: 1.00 (NS)  Medium demand/low control: 1.10 (S)  Low demand/high control: 0.93 (S)  Low demand/medium control: 1.09 (S)  Low demand/low control: 1.42 (S)  High increasing SA/DP trajectory, men  High demand/high control: 0.74 (S)  High demand/medium control: 0.80 (S)  High demand/low control: 1.11 (S)  Medium demand/high control: 0.93 (S)  Medium demand/medium control: 1.00 (NS)  Medium demand/low control: 1.08 (S)  Low demand/high control: 1.23 (S)  Low demand/medium control: 1.23 (S)  Low demand/low control: 1.13 (S) | Gender stratified analyses.  Analyses were adjusted for all covariates. ORs were presented in a graph with no specific numbers for the 95% CI. No unadjusted analyses were reported. |
| Days lost due to SA and DP | Shiri et al. 2021 | Biomechanical: heavy lifting or pulling or pushing heavy loads, back rotations, awkward working positions  Psychosocial:  job strain (high demands/low control) | Personal factors:  gender, education  Occupational factors:  occupational class  Lifestyle factors:  smoking, BMI, leisure-time physical activity, binge drinking  Health-related factors:  long-standing illness, common mental disorder, number of pain sites | On average, during 10 years of follow-up 316 calendar days (220 working days) were lost; 44% due to SA and 56% due to DP.  Adjusted model (IRR (95% CI)):  All  heavy lifting, or pulling or pushing heavy loads: 1.35 (1.10, 1.65)  back rotations: 0.99 (0.82, 1.21)  awkward working positions: 1.24 (1.01, 1.53)  high job strain: 0.85 (0.72–0.997)  Women  heavy lifting, or pulling or pushing heavy loads: 1.31 (1.06, 1.61)  back rotations: 0.91 (0.74, 1.12)  awkward working positions: 1.45 (1.16, 1.81)  high job strain: 0.82 (0.69, 0.98) | Associations were tested in two models: (1) unadjusted and (2) adjusted for all variables.  The unadjusted results are presented both for all and stratified by gender, whilst the full model is presented for all and for only women (81% of the sample).  Unadjusted results (whole population):  heavy lifting, or pulling or pushing heavy loads: 2.23 (1.91, 2.60)  back rotations: 1.96 (1.69, 2.26)  awkward working positions: 2.00 (1.71, 2.32)  high job strain: 1.34 (1.14, 1.59)  Men:  heavy lifting, or pulling or pushing heavy loads: 2.13 (1.29, 3.53)  back rotations: 1.97 (1.29, 2.98)  awkward working positions: 1.36 (0.91, 2.02)  high job strain: 2.00 (1.26, 3.17)  Women:  heavy lifting, or pulling or pushing heavy loads: 2.25 (1.91, 2.63)  back rotations: 1.96 (1.67, 2.28)  awkward working positions: 2.13 (1.82, 2.50)  high job strain: 1.24 (1.04, 1.48) |
| *Working life patterns based on multiple states, trajectories* | | |  |  |  |
| Clusters of working life sequencies | Gémes et al. 2023 | Biomechanical:  physically strenuous job  Psychosocial:  mentally strenuous job, monotonous job, possibility to learn new things at work, hectic work schedule  Physical work environment: noise at work | Personal factors:  age, education, participants/both parents born outside Sweden, nationality, living area, family situation, economic hardship  Occupational factors:  working hours, previous work accident  Lifestyle factors:  daily smoking overweight/obesity  Health-related factors:  previous SA/DP, long-term illness or health problems, self-rated health | 1 284 unique future labour market sequences identified, which were grouped into five clusters: Active (65.1), Parental-leave periods (12.7%), Unemployment & SA/ DP periods (11%), SA/DP periods (7.7%), and Retirement (3.5%). Active was used as the reference group.  Adjusted results (OR (95% CI)):  Unemployment & SA/DP, men  physically strenuous job: 1.0 (0.8, 1.3)  mentally strenuous job: 1.1 (0.9, 1.3)  monotonous job: 1.4 (1.2, 1.8)  possibility to learn new things at work: 0.8 (0.6, 1.0)  hectic work schedule: 0.8 (0.6, 0.9)  noise at work: 0.7 (0.6, 0.9)  Parental-leave periods, men  physically strenuous job: 1.0 (0.5, 1.9)  mentally strenuous job: 1.9 (0.5, 1.5)  monotonous job: 1.4 (0.9, 2.3)  possibility to learn new things at work: 0.7 (0.4, 1.2)  hectic work schedule: 1.2 (0.7, 2.0)  noise at work: 0.5 (0.3, 0.9)  SA/DP periods, men  physically strenuous job: 1.4 (1.0, 2.1)  mentally strenuous job: 1.1 (0.8, 1.5)  monotonous job: 1.1 (0.8, 1.4)  possibility to learn new things at work: 0.9 (0.7, 1.2)  hectic work schedule: 1.1 (0.8, 1.5)  noise at work: 0.9 (0.7, 1.3)  Retirement, men  physically strenuous job: 0.9 (0.6, 1.3)  mentally strenuous job: 1.2 (0.8, 1.6)  monotonous job: 1.5 (1.0, 2.1)  possibility to learn new things at work: 0.9 (0.6, 1.3)  hectic work schedule: 0.9 (0.6, 1.2)  noise at work: 0.8 (0.5, 1.1)  Unemployment & SA/ DP, women  physically strenuous job: 0.8 (0.6, 1.1)  mentally strenuous job: 0.8 (0.6, 1.0)  monotonous job: 1.1 (0.9, 1.4)  possibility to learn new things at work: 0.8 (0.6, 1.0)  hectic work schedule: 1.0 (0.8, 1.3)  noise at work: 1.1 (0.8, 1.4)  Parental-leave periods, women  physically strenuous job: 0.9 (0.7, 1.2)  mentally strenuous job: 1.1 (0.9, 1.3)  monotonous job: 1.0 (0.8, 1.3)  possibility to learn new things at work: 1.2 (1.0, 1.4)  hectic work schedule: 1.2 (1.0, 1.5)  noise at work: 0.8 (0.7, 1.1)  SA/DP periods, women  physically strenuous job: 0.9 (0.7, 1.3)  mentally strenuous job: 1.2 (1.0, 1.6)  monotonous job: 1.2 (0.9, 1.6)  possibility to learn new things at work: 0.9 (0.7, 1.2)  hectic work schedule: 1.2 (0.9, 1.6)  noise at work: 0.9 (0.7, 1.2)  Retirement, women  physically strenuous job: 0.8 (0.5, 1.3)  mentally strenuous job: 1.0 (0.7, 1.4)  monotonous job: 1.2 (0.8, 1.7)  possibility to learn new things at work: 0.9 (0.6, 1.3)  hectic work schedule: 1.0 (0.6, 1.5)  noise at work: 1.1 (0.7, 1.7) | Gender-stratified analyses.  No unadjusted analyses were reported. |
| Work and disability trajectories | Harrati et al. 2019 | Physical work environment: particulate matter 2.5 (PM 2.5), categorized into quartiles. | Personal factors:  age, sex, ethnicity  Occupational factors:  tenure (total time employed), hourly versus salaried work, income, work in smelting plant versus fabrication  Health-related factors:  in- and outpatient health care visits due to seven chronic diseases (depression, ischemic heart disease, hypertension, arthritis, asthma and musculoskeletal conditions), health risk score (general health) | Employees were clustered into four groups: regular work (54% of sample, reference group), short STD (22%), disruptive work (22%), ever LTD (1.65%).  Adjusted analyses (RRR):  Short STD  Quartile 1 used as reference  Quartile 2: 1.45 (S)  Quartile 3: 1.52 (S)  Quartile 4: 1.47 (S)  Disruptive work  Quartile 2: 1.86 (S)  Quartile 3: 2.15 (S)  Quartile 4: 1.99 (S)  Ever LTD  Quartile 2: 1.36 (NS)  Quartile 3: 1.61 (NS)  Quartile 4: 0.68 (NS) | Analyses were adjusted for all variables; no unadjusted analyses were reported. RRRs were reported with robust standard errors and indications of statistical significance; no CIs were reported. |
| Trajectories of labour market attachment | Hartikainen et al. 2023 | Biomechanical: physically heavy work  Psychosocial factors: job control | Personal factors:  age, gender, education, income  Occupational factors:  employment sector  Health-related factors:  chronic diseases (musculoskeletal, mental, nervous, circulatory, neoplasms, injuries), start year of part-time SA. | Five trajectories identified: sustained work (40%), slightly reduced work (32%), partial work disability (12%), full work disability (8%), other non-employed (8%).  Adjusted results (OR (95% CI)):  Sustained work  physically heavy work: 0.85 (0.76, 0.94)  low job control: 0.95 (0.87, 1.06)  Slightly reduced work  physically heavy work: 1.17 (1.06, 1.23)  low job control: 1.04 (0.94, 1.15)  Partial work disability  physically heavy work: 1.02 (0.88, 1.18)  low job control: 1.07 (0.93, 1.23)  Full work disability  physically heavy work: 0.98 (0.82, 1.18)  low job control: 0.86 (0.73, 1.03)  Other non-employed  physically heavy work: 1.00 (0.84, 1.19)  low job control: 1.09 (0.92, 1.30) | Associations were tested in two models: (1) age-adjusted and (2) adjusted for all variables.  In the age adjusted model, physically heavy work was associated with reduced risk of membership in sustained work group and increased risk of membership in slightly reduced work and partial work disability groups.  Low job control was associated with reduced risk of membership in sustained work group and increased risk of membership in slightly reduced work, full work disability groups and other non-employed groups. |
| Work participation trajectories | Leinonen et al. 2019 | Biomechanical: heavy physical work, kneeling/squatting, repetitive hand movements  Psychosocial factors: job strain, monotonous work.  Exposures were dichotomized. | Personal factors:  age, gender, region of residence, education  Occupational factors:  industrial sector, employment sector  Health-related factors:  disease group of rehabilitation, start year of rehabilitation, duration of rehabilitation | Four trajectory groups of work participation (share of time at work) identified: High-Resumed (35.6% of population), High-to-Negligible (20.7%), Medium-Resumed (25.5%), and Longstanding Negligible (18.3%). The High-Resumed trajectory was used as the reference group.  Adjusted analyses (RRR (95% CI)):  High-to-Negligible  heavy physical work: 1.08 (0.76, 1.53) (low), 1.14 (0.78, 1.68) (high)  kneeling/squatting: 0.92 (0.69, 1.20) (low), 0.97 (0.70, 1.33) (high)  repetitive hand movement: 0.99 (0.96, 1.03)  monotonous work: 1.12 (0.92, 1.36)  active job: 1.18 (0.90, 1.56)  passive job: 0.92 (0.74, 1.15)  high strain job: 1.18 (0.91, 1.53)  Medium-Resumed  heavy physical work: 0.88 (0.63, 1.23) (low), 0.80 (0.55, 1.16) (high)  kneeling/squatting: 1.27 (0.95, 1.69) (low), 1.39 (1.02, 1.90) (high)  repetitive hand movement: 1.00 (0.96, 1.03)  monotonous work: 1.18 (0.98, 1.43)  active job: 1.05 (0.81, 1.35)  passive job: 0.91 (0.75, 1.12)  high strain job: 1.15 (0.90, 1.47)  Longstanding Negligible  heavy physical work: 1.36 (0.90, 2.07) (low), 1.19 (0.76, 1.87) (high)  kneeling/squatting: 1.06 (0.76, 1.48) (low), 1.24 (0.87, 1.76) (high)  repetitive hand movement: 1.02 (0.98, 1.06)  monotonous work: 1.00 (0.81, 1.24)  active job: 1.08 (0.79, 1.48)  passive job: 1.06 (0.83, 1.35)  high strain job: 1.35 (1.02, 1.80) | Associations were tested in two models: (1) age- and gender-adjusted and (2) adjusted for all variables.  In the age- and gender-adjusted model, heavy physical work (both low and high) was associated with increased risk of membership in the Longstanding Negligible trajectory. Kneeling/squatting (both low and high) was associated with increased risk of membership in Medium-Resumed and Longstanding Negligible trajectories.  Repetitive hand movement and monotonous work were associated with increased risk of membership in the High-to-Negligible trajectory.  High strain job was associated with increased risk of membership in High-to-Negligible and Longstanding Negligible trajectories. |
| *Working life patterns based on multiple states, cumulative time* | | | | | |
| WLE from age 50 | Chungkham et al., 2024 | Psychosocial: job strain | Personal factors: education  Occupational factors: occupation | Total WLE at age 50, men  No strain: 13.44 (13.24, 13.65)  Strain: 12.72 (12.34, 12.99)  Full-time WLE at age 50, men  No strain: 11.68 (11.49, 11.84)  Strain: 11.13 (10.79, 11.37)  Part-time WLE at age 50, men  No strain: 1.76 (1.63, 1.90)  Strain: 1.60 (1.39, 1.81)  Total WLE at age 50, women  No strain: 12.82 (12.64, 12.97)  Strain: 12.16 (11.86, 12.43)  Full-time WLE at age 50, women  No strain: 11.00 (10.84, 11.13)  Strain: 10.50 (10.24, 10.72)  Part-time WLE at age 50, women  No strain: 2.00 (1.87, 2.13)  Strain: 1.91 (1.67, 2.13) | Gender-stratified analyses. |
| HWLE at age 50 years | Lynch et al. 2024 | Psychosocial factors: lack of autonomy at work, inadequate social support at work | Personal factors:  age, sex  Health-related factors:  osteoarthritis | Lack of autonomy was associated with an increased risk of transition from healthy and working (HWLE) to not healthy and/or not working.  Adjusted analyses (HWLE in years (95% CI)):  HWLE at age 50:  overall: 9.03 (8.78, 9.29)  no autonomy at work: 7.67 (7.22, 8.12)  has autonomy at work: 9.50 (9.20, 9.79)  no support at work: 7.86 (7.46, 8.27)  has support at work: 9.52 (9.22, 9.82)  Lack of autonomy in combination with sex and osteoarthritis (OA) status:  male, no OA, has autonomy at work: 10.79 (10.36, 11.22)  male, no OA, no autonomy at work: 8.78 (8.18, 9.38)  male, with OA, has autonomy at work: 8.63 (7.90, 9.35)  male, with OA, no autonomy at work: 6.89 (6.01, 7.77)  female, no OA, has autonomy at work: 9.20 (8.79, 9.60)  female, no OA, no autonomy at work: 7.42 (6.95, 7.91)  female, with OA, has autonomy at work: 7.22 (6.71, 7.73)  female, with OA, no autonomy at work: 5.70 (5.17, 6.22)  Lack of support at work in combination with sex and osteoarthritis (OA) status:  male, no OA, has support at work: 10.89 (10.45, 11.34)  male, no OA, no support at work: 9.01 (8.49, 9.53)  male, with OA, has support at work: 8.70 (7.96, 9.45)  male, with OA, no support at work: 7.06 (6.27, 7.85)  female, no OA, has support at work: 9.20 (8.80, 9.61)  female, no OA, no support at work: 7.51 (7.04, 7.97)  female, with OA, has support at work: 7.21 (6.71, 7.72)  female, with OA, no support at work: 5.76 (5.26, 6.27) | Models adjusted for age. Gender-stratified analyses in addition to total. |
| WLE and WYL at age 30, 40 and 50 years | Pedersen et al. 2020 | Physical work demands summary index score categorized into tertiles: low, middle and high |  | Individuals with high physical work demands spent significantly less time in work.  Model results (WLE in years (95% CI)):  WLE at age 30, men  Low demands: 33.89 (33.82, 33.97)  Middle demands: 32.34 (32.20, 32.47)  High demands: 31.87 (31.71, 32.04)  WYL due to SA at age 30, men  Low demands: 0.44 (0.40, 0.49)  Middle demands: 0.95 (0.86, 1.03)  High demands: 1.48 (1.36, 1.60)  WYL due to disability pension at age 30, men  Low demands: 0.09 (0.07, 0.11)  Middle demands: 0.40 (0.35, 0.45)  High demands: 0.21 (0.17, 0.26)  WYL due to unemployment at age 30, men  Low demands: 0.36 (0.32, 0.41)  Middle demands: 0.95 (0.86, 1.04)  High demands: 1.05 (0.95, 1.15)  WLE at age 30, women  Low demands: 32.67 (32.56, 32.78)  Middle demands: 31.07 (30.91, 31.23)  High demands: 29.62 (29.41, 29.84)  WYL due to SA at age 30, women  Low demands: 0.99 (0.91, 1.07)  Middle demands: 1.53 (1.42, 1.64)  High demands: 1.90 (1.77, 2.03)  WYL due to disability pension at age 30, women  Low demands: 0.29 (0.25, 0.33)  Middle demands: 0.88 (0.80, 0.95)  High demands: 0.93 (0.84, 1.02)  WYL due to unemployment at age 30, women  Low demands: 0.56 (0.50, 0.62)  Middle demands: 0.94 (0.86, 1.03)  High demands: 1.90 (1.75, 2.06)  WLE at age 40, men  Low demands: 24.22 (24.18, 24.27)  Middle demands: 23.10 (23.00, 23.19)  High demands: 22.72 (22.61, 22.84)  WYL due to SA at age 40, men  Low demands: 0.36 (0.32, 0.39)  Middle demands: 0.76 (0.70, 0.82)  High demands: 1.19 (1.10, 1.28)  WYL due to disability pension at age 40, men  Low demands: 0.07 (0.06, 0.09)  Middle demands: 0.32 (0.28, 0.35)  High demands: 0.17 (0.13, 0.20)  WYL due to unemployment at age 40, men  Low demands: 0.24 (0.21, 0.27)  Middle demands: 0.63 (0.57, 0.69)  High demands: 0.70 (0.63, 0.77)  WLE at age 40, women  Low demands: 23.64 (23.57, 23.71)  Middle demands: 22.58 (22.48, 22.68)  High demands: 21.62 (21.49, 21.76)  WYL due to SA at age 40, women  Low demands: 0.73 (0.68, 0.78)  Middle demands: 1.13 (1.06, 1.20)  High demands: 1.41 (1.33, 1.50)  WYL due to disability pension at age 40, women  Low demands: 0.19 (0.16, 0.21)  Middle demands: 0.56 (0.52, 0.61)  High demands: 0.60 (0.55, 0.66)  WYL due to unemployment at age 40, women  Low demands: 0.35 (0.31, 0.39)  Middle demands: 0.59 (0.54, 0.65)  High demands: 1.20 (1.11, 1.30)  WLE at age 50, men  Low demands: 14.48 (14.45, 14.51)  Middle demands: 13.73 (13.67, 13.79)  High demands: 13.46 (13.38, 13.54)  WYL due to SA at age 50, men  Low demands: 0.25 (0.23, 0.28)  Middle demands: 0.54 (0.50, 0.58)  High demands: 0.84 (0.78, 0.90)  WYL due to disability pension at age 50, men  Low demands: 0.04 (0.03, 0.05)  Middle demands: 0.20 (0.17, 0.22)  High demands: 0.11 (0.08, 0.13)  WYL due to unemployment at age 50, men  Low demands: 0.16 (0.14, 0.18)  Middle demands: 0.42 (0.38, 0.46)  High demands: 0.47 (0.42, 0.51)  WLE at age 50, women  Low demands: 14.20 (14.15, 14.24)  Middle demands: 13.58 (13.52, 13.65)  High demands: 12.98 (12.89, 13.07)  WYL due to SA at age 50, women  Low demands: 0.45 (0.42, 0.49)  Middle demands: 0.70 (0.66, 0.75)  High demands: 0.88 (0.83, 0.94)  WYL due to disability pension at age 50, women  Low demands: 0.09 (0.08, 0.11)  Middle demands: 0.28 (0.25, 0.31)  High demands: 0.30 (0.27, 0.33)  WYL due to unemployment at age 50, women  Low demands: 0.23 (0.20, 0.25)  Middle demands: 0.38 (0.34, 0.42)  High demands: 0.77 (0.71, 0.84) | Gender-stratified analyses. |
| ELMA | Pedersen et al. 2022 | Physical work demands ergonomic index score categorized in to low, moderate, high and very high | Personal factors:  education  Occupational factors:  working time arrangement (part-time vs. full time), employment sector  Lifestyle factors:  BMI, smoking, leisure-time physical activity  Health-related factors:  diseases (depression, asthma, diabetes, atherosclerosis or blood clot in the heart, blood clot in the brain (cerebral hemorrhage), cancer, back disease, migraine, or other long-term disease), symptoms of depression  Other factors:  number of survey waves | Results were presented as change in ELMA compared to the reference category (low physical work demands).  ELMA in days (95% CI)  Expected days at work, men, age 18-39  Low demands: 677.1 (672.2, 682.0)  Moderate demands: + 8.6 (1.7, 15.6)  High demands: + 7.5 (0.6, 14.5)  Very high demands: - 8.3 (-15.3, -1.4)  Expected days lost due to SA, men, age 18-39  Low demands: 14.6 (12.0, 17.3)  Moderate demands: - 3.0 (-6.7, 0.7)  High demands: + 0.8 (-2.9, 4.5)  Very high demands: + 8.6 (4.9, 12.3)  Expected days lost due to unemployment, men, age 18-39  Low demands: 5.4 (3.4, 7.4)  Moderate demands: - 0.2 (-3.0, 2.6)  High demands: + 2.3 (-0.5, 5.1)  Very high demands: + 5.2 (2.4, 8.0)  Expected days at work, women, age 18-39  Low demands: 616.5 (611.1, 622.0)  Moderate demands: + 8.7 (1.0, 16.4)  High demands: - 4.1 (-11.8, 3.5)  Very high demands: + 7.7 (0.0, 15.4)  Expected days lost due to SA, women, age 18-39  Low demands: 32.8 (29.6, 36.0)  Moderate demands: - 0.7 (-5.2, 3.8)  High demands: + 0.2 (-4.3, 4.7)  Very high demands: - 3.8 (-8.3, 0.7)  Expected days lost due to unemployment, women, age 18-39  Low demands: 9.2 (7.5, 10.9)  Moderate demands: - 0.0 (-2.4, 2.4)  High demands: + 0.3 (-2.1, 2.7)  Very high demands: + 4.3 (1.9, 6.7)  Expected days at work, men, age 40-49  Low demands: 704.8 (700.9, 708.6)  Moderate demands: - 3.5 (-9.0, 2.0)  High demands: - 20.7 (-26.1, -15.2)  Very high demands: - 26.0 (-31.5, -20.6)  Expected days lost due to SA, men, age 40-49  Low demands: 9.6 (6.6, 12.7)  Moderate demands: + 5.8 (1.5, 10.1)  High demands: + 19.2 (14.9, 23.5)  Very high demands: + 23.3 (19.0, 27.6)  Expected days lost due to unemployment, men, age 40-49  Low demands: 2.8 (1.6, 4.1)  Moderate demands: + 1.3 (-0.5, 3.1)  High demands: + 6.9 (5.1, 8.7)  Very high demands: + 3.8 (2.0, 5.6)  Expected days at work, women, age 40-49  Low demands: 688.7 (684.7, 692.7)  Moderate demands: - 15.7 (-21.4, -10.0)  High demands: - 23.0 (-28.7, -17.3)  Very high demands: - 34.5 (-40.1, -28.8)  Expected days lost due to SA, women, age 40-49  Low demands: 27.2 (24.1, 30.3)  Moderate demands: + 8.2 (3.8, 12.6)  High demands: + 11.3 (6.9, 15.8)  Very high demands: + 26.3 (21.8, 30.7)  Expected days lost due to unemployment, women, age 40-49  Low demands: 6.0 (4.6, 7.4)  Moderate demands: + 0.8 (-1.2, 2.8)  High demands: + 0.8 (-1.2, 2.8)  Very high demands: + 4.0 (2.0, 6.0)  Expected days at work, men, age 50-64  Low demands: 671.0 (666.9, 675.0)  Moderate demands: - 1.6 (-7.3, 4.2)  High demands: - 23.2 (-29.0, -17.5)  Very high demands: - 21.7 (-27.4, -15.9)  Expected days lost due to SA, 50-64 yrs. old men  Low demands: 18.5 (15.9, 21.0)  Moderate demands: + 3.7 (0.1, 7.3)  High demands: + 20.2 (16.6, 23.8)  Very high demands: + 12.1 (8.5, 15.7)  Expected days lost due to unemployment, 50-64 yrs. old men  Low demands: 3.6 (2.3, 4.9)  Moderate demands: + 0.7 (-1.1, 2.5)  High demands: + 5.2 (3.4, 7.1)  Very high demands: + 9.4 (7.5, 11.2)  Expected days at work, women, age 50-64  Low demands: 654.1 (650.0, 658.3)  Moderate demands: - 2.8 (-8.6, 3.0)  High demands: - 10.1 (-16.0, -4.3)  Very high demands: - 21.1 (-26.9, -15.2)  Expected days lost due to SA, women, age 50-64  Low demands: 27.8 (24.9, 30.7)  Moderate demands: + 13.4 (9.4, 17.5)  High demands: + 12.4 (8.3, 16.5)  Very high demands: + 23.1 (19.1, 27.2)  Expected days lost due to unemployment, women, age 50-64  Low demands: 5.0 (3.7, 6.3)  Moderate demands: + 3.7 (1.9, 5.5)  High demands: + 5.3 (3.5, 7.1)  Very high demands: + 5.3 (3.5, 7.1) | Gender- and age group-stratified analyses. Covariates were included as weights. |
| WLE and WYL at age 50 years | Schram et al. 2021 | Biomechanical: heavy physical work, heavy lifting, kneeling/squatting, working with hands above shoulder level, awkward posture  Number of factors reported used.  Exposures were dichotomized. |  | WLE, men  no factors: 10.44 (10.43, 10.46)  1-3 factors: 10.03 (10.01, 10.05)  4-5 factors: 9.54 (9.50, 9.58)  WYL due to time-restricted  work disability, men  no factors: 0.33 (0.33, 0.34)  1-3 factors: 0.43 (0.43, 0.44)  4-5 factors: 0.56 (0.55, 0.57)  WYL due to disability retirement, men  no factors: 0.50 (0.49, 0.52)  1-3 factors: 0.70 (0.68, 0.71)  4-5 factors: 0.95 (0.93, 0.98)  WYL due to unemployment, men  no factors: 0.58 (0.57, 0.59)  1-3 factors: 0.67 (0.67, 0.68)  4-5 factors: 0.76 (0.75, 0.78)  WLE, women  no factors: 10.42 (10.40, 10.44)  1-3 factors: 9.98 (9.95, 10.00  4-5 factors: 9.44 (9.41, 9.48)  WYL due to time-restricted  work disability, women  no factors: 0.45 (0.45, 0.46)  1-3 factors: 0.60 (0.59, 0.61)  4-5 factors: 0.78 (0.76, 0.79)  WYL due to disability retirement, women  no factors: 0.47 (0.45, 0.48)  1-3 factors: 0.69 (0.67, 0.71)  4-5 factors: 1.00 (0.96, 1.03)  WYL due to unemployment, women  no factors: 0.61 (0.60, 0.62)  1-3 factors: 0.63 (0.62, 0.64)  4-5 factors: 0.63 (0.61, 0.65) | Gender-stratified analyses.  Analyses were repeated within occupational classes (manual workers, lower non-manual, upper non-manual, and self-employed).  There were differences in WLE between the most advantaged group (upper non-manual employees with 0 exposures) and the most disadvantaged group (manual workers with four or five exposures; 1.56 years for men, 1.59 years for women). |
| WYL due to involuntary (disability benefits and unemployment) and voluntary (economic inactivity and early retirement) exit at age 50 | Schram et al. 2022 | Biomechanical (physical workload) summary score (force exertion, static load (standing, posture and kneeling), vibration)  Psychosocial:  psychological job demands, autonomy, emotional demands  Factors were categorised into quartiles and further dichotomized |  | WYL due to involuntary exit, low education  low physical workload: 1.84 (1.55, 2.12)  high physical workload: 1.93 (1.65, 2.20)  low job demands: 1.81 (1.56, 2.08)  high job demands: 2.15 (1.83, 2.48)  high autonomy: 1.80 (1.55, 2.08)  low autonomy: 2.01 (1.72, 2.31)  low emotional job demands: 1.88 (1.63, 2.15)  high emotional job demands: 2.11 (1.75, 2.50)  WYL due to involuntary exit, intermediate education  low physical workload: 1.60 (1.46, 1.76)  high physical workload: 1.68 (1.51, 1.87)  low job demands: 1.56 (1.42, 1.70)  high job demands: 1.87 (1.66, 2.08)  high autonomy: 1.58 (1.44, 1.71)  low autonomy: 1.74 (1.55, 1.94)  low emotional job demands: 1.62 (1.48, 1.75)  high emotional job demands: 1.82 (1.57, 2.08)  WYL due to involuntary exit, high education  low physical workload: 1.40 (1.31, 1.48)  high physical workload: 1.46 (1.31, 1.62)  low job demands: 1.34 (1.27, 1.43)  high job demands: 1.61 (1.47, 1.77)  high autonomy: 1.38 (1.29, 1.46)  low autonomy: 1.51 (1.37, 1.66)  low emotional job demands: 1.38 (1.31, 1.47)  high emotional job demands: 1.56 (1.38, 1.74)  WYL due to voluntary exit, low education  low physical workload: 1.61 (1.44, 1.78)  high physical workload: 1.59 (1.41, 1.75) low job demands: 1.63 (1.47, 1.79)  high job demands: 1.56 (1.36, 1.75)  high autonomy: 1.60 (1.44, 1.74)  low autonomy: 1.64 (1.47, 1.83)  low emotional job demands: 1.61 (1.45, 1.76)  high emotional job demands: 1.75 (1.53, 1.97)  WYL due to voluntary exit, intermediate education  low physical workload: 1.63 (1.52, 1.73)  high physical workload: 1.61 (1.48, 1.73)  low job demands: 1.65 (1.55, 1.74)  high job demands: 1.58 (1.46, 1.72)  high autonomy: 1.62 (1.52, 1.72)  low autonomy: 1.65 (1.53, 1.78)  low emotional job demands: 1.62 (1.52, 1.72)  high emotional job demands: 1.76 (1.59, 1.92)  WYL due to voluntary exit, high education  low physical workload: 1.64 (1.57, 1.71)  high physical workload: 1.63 (1.52, 1.75)  low job demands: 1.66 (1.59, 1.73)  high job demands: 1.60 (1.51, 1.72)  high autonomy: 1.63 (1.57, 1.71)  low autonomy: 1.66 (1.55, 1.77)  low emotional job demands: 1.62 (1.55, 1.68)  high emotional job demands: 1.76 (1.63, 1.90) | Education-stratified analyses. |
